# Machine learning prediction of sudden cardiac death incorporating multiple lipid markers: evidence from the Taiwan Chin Shan community cohort

**DOI:** 10.1186/s12944-026-02984-5

**Published:** 2026-05-30

**Authors:** Yun-Yu Chen, Yang Chen, Kuo-Liong Chien, Qinkai Yu, Yu-Cheng Hsieh, Fang-Yi Chen, Yenn-Jiang Lin, Gregory Y. H. Lip, Shih-Ann Chen

**Affiliations:** 1 Department of Medical Research, Taichung Veterans General Hospital, Taichung, Taiwan; 2 Cardiovascular Center, Taichung Veterans General Hospital, Taichung, Taiwan; 3 Department of Post-Baccalaureate Medicine, National Chung Hsing University School of Medicine, Taichung, Taiwan; 4 Heart Rhythm Center, Division of Cardiology, Department of Medicine, Taipei Veterans General Hospital, Taipei, Taiwan; 5 Liverpool Centre for Cardiovascular Science, University of Liverpool, Liverpool John Moores University and Liverpool Heart & Chest Hospital, Liverpool, UK; 6 Institute of Epidemiology and Preventive Medicine College of Public Health, National Taiwan University, Taipei, Taiwan; 7 Computer Science Department, University of Exeter, Exeter, UK; 8 School of Medicine, National Yang Ming Chiao Tung University, Taipei, Taiwan; 9 Department of Internal Medicine, Taichung Veterans General Hospital, Taichung, Taiwan; 10 Danish Center for Health Services Research, Department of Clinical Medicine, Aalborg University, Aalborg, Denmark; 11 Department of Cardiology, Lipidology and Internal Medicine, Medical University of Bialystok, Bialystok, Poland

**Keywords:** Community cohort, Machine learning, Prediction model, Risk factors, Sudden cardiac death

## Abstract

**Background:**

Sudden cardiac death (SCD) is a major contributor to cardiovascular mortality, but reliable long-term risk prediction in community-based populations remains limited. Machine learning (ML) offers potential advantages, yet its application to SCD prediction remains comparatively limited, particularly in community-based populations.

**Methods:**

We used data from the Chin-Shan Community Cardiovascular Cohort (CCCC), a prospective community-based cohort in Taiwan enrolling adults aged ≥ 35 years from 14 villages. Participants were geographically partitioned into training/internal validation and independent external validation cohorts. After feature preselection using the Boruta algorithm, six ML models were developed (Light Gradient Boosting Machine, Random Forest [RF], Logistic Regression, Support Vector Machine, Multilayer Perceptron, and K-Nearest Neighbours), with class imbalance addressed using appropriate techniques. Model performance was evaluated using discrimination and classification metrics, including the area under the curve (AUC), positive predictive value, and negative predictive value (NPV). The optimal model was interpreted using SHapley Additive exPlanations and implemented as a web-based risk calculator.

**Results:**

A total of 3,172 participants were included (median age [IQR]: 54.9 [45.8–64.0] years; 47.4% male), with 74 SCD events observed over a median follow-up of 15.9 years (IQR, 13.1–16.9 years). Ten non-collinear predictors were preselected, and six ML models were developed and validated. The RF showed the highest discrimination in internal (AUC: 0.824) and external validation (AUC: 0.815) and was the only model to significantly outperform the Framingham Risk Score. The RF demonstrated consistently high NPVs (> 99%) in both internal and external validation cohorts and was implemented as a web-based risk calculator.

**Conclusions:**

We developed and externally validated a RF model for long-term SCD risk prediction with high NPV, supporting its potential utility for identifying low-risk individuals in community settings, pending further validation. The model has been implemented as an online risk calculator, with further validation in larger and diverse populations warranted.

**Supplementary Information:**

The online version contains supplementary material available at 10.1186/s12944-026-02984-5.

## Introduction

Sudden cardiac death (SCD) represents a major and enduring global public health challenge, accounting for a substantial proportion of cardiovascular-related mortality worldwide [1]. Despite advances in cardiovascular prevention and treatment, the proportion of SCD among cardiovascular deaths remains considerable and varies markedly across different regions and populations [2]. Although estimates vary, SCD is consistently recognised as a major component of cardiovascular mortality across diverse populations, with observed differences largely attributable to risk factors such as age distribution [3], and lipid-related risk profiles [4, 5].

Traditional risk stratification approaches, largely based on clinical parameters and conventional statistical methods, have demonstrated limited predictive accuracy for sudden cardiac death, resulting in both underestimation and overestimation of individual risk [3, 6, 7]. Consequently, there is an urgent need for more accurate prediction models to improve early identification and targeted intervention in high-risk individuals. By leveraging large-scale datasets and advanced algorithms, machine learning (ML) can capture complex, non-linear relationships that are often missed by traditional statistical models [8, 9]. As a result, ML-based models offer the potential to enhance risk stratification by integrating a broad range of clinical and lifestyle factors, enabling more precise and individualised risk assessment [10].

While ML approaches have been increasingly applied to predict cardiovascular outcomes such as stroke, myocardial infarction, and cardiac death [8, 10, 11], their application to sudden cardiac death prediction remains less extensively studied, particularly in prospective community-based cohorts. Previous work has developed conventional point-based risk scores for SCD prediction, but such approaches rely on pre-specified predictor selection and may be less suited to capturing complex, non-linear relationships among multidimensional risk factors [3]. Recognising this gap, we aimed to develop and validate an ML-based model for SCD risk prediction using data from the Chin-Shan Community Cardiovascular Cohort (CCCC), a prospective, multi-region, community-based cohort in Taiwan [3, 12].

## Methods

### Study design and setting

An established community-based cohort study in Taiwan, the CCCC, was utilized for this study. In 1990 and 1991, the CCCC enrolled participants aged ≥ 35 years from 14 geographically defined villages in the Chin-Shan area. This cohort was originally established to investigate cardiovascular health and disease outcomes in the general population. A broad spectrum of baseline clinical data was meticulously gathered, encompassing demographic details, medical history, lifestyle determinants, and cardiovascular risk factors [3, 12, 13]. This research was reviewed and approved by the Institutional Review Board of National Taiwan University Hospital (IRB Number: 2011003001R), following the Good Clinical Practice Guidelines. Both verbal and written informed consent were obtained from all adult participants involved in the study.

### Study population and outcome

The study population included participants with complete baseline blood biomarker data available for analysis. Mortality outcomes were identified using government death certificates. Participants were prospectively followed for mortality outcomes until 2005. The primary outcome was SCD, defined as a sudden, unexpected, non-traumatic loss of cardiac function and vital signs without preceding complaints or illness, or death occurring within one hour of the onset of complaints. Victims who were found dead were also classified as SCD if they had been seen alive and well within 24 h of the incident. Intoxicated or chronically ill patients experiencing circulatory arrest were excluded. SCD events were ascertained through systematic follow-up procedures, including review of government death certificates, medical records, and relevant clinical documentation, together with interviews with family members, witnesses, and the physician in charge when available. These interviews were conducted within one month after death and were reviewed by three investigating physicians, with particular attention to the mode of death and the symptoms or signs preceding death. Among the 74 adjudicated SCD events, 38 were witnessed SCD events, accounting for 51.4% of all SCD cases.

### Cohort partitioning

To ensure robust model development and validation while minimizing information leakage, a geographically structured data partitioning strategy was adopted. The 14 villages included in the CCCC were first randomly assigned numerical identifiers. Participants from villages numbered 1 to 10 were used for model development and internal validation. Participants from the remaining villages (villages 11 to 14) were reserved as an independent external validation cohort, which was not involved in any stage of model training or threshold optimization.

### Data collection

Baseline data were collected at study entry using standardized protocols by trained personnel. Information on demographic characteristics, medical history, medication use, and lifestyle factors was obtained through structured questionnaires administered during face-to-face interviews. Anthropometric measurements, including height and weight, were obtained using calibrated instruments, and body mass index was calculated accordingly. Blood pressure was measured in the seated position after at least five minutes of rest using a mercury sphygmomanometer in the Chin-Shan community, northern Taiwan, with the average of two measurements used for analysis. Baseline 12-lead electrocardiograms were obtained using standardized recording protocols and independently interpreted by two experienced physicians for predefined electrocardiographic abnormalities according to established criteria. ECG measurements were physician-reviewed using standard reference values or deviations, and ECG abnormalities were assessed based on serial ECG examinations obtained during biennial follow-up assessments. LVH was defined using both echocardiographic and electrocardiographic criteria. Echocardiographic LVH was determined according to sex-specific left ventricular mass index thresholds, defined as LV mass index ≥ 132 g/m² in men and ≥ 109 g/m² in women. ECG-defined LVH was determined using the Sokolow–Lyon voltage criterion, calculated as the S-wave amplitude in lead V1 plus the greatest R-wave amplitude in lead V5 or V6 > 35 mm. LVH was treated as a binary predictor in model development. Discrepancies were resolved through adjudication by a third senior physician, consistent with the cohort’s cardiovascular outcome validation procedures. All collected data were systematically reviewed, verified, and curated to ensure completeness and internal consistency prior to statistical analysis and model development [12, 13].

Venous blood samples were collected after a 12-hour overnight fast, immediately refrigerated, and transported within 6 h to National Taiwan University Hospital, where serum was separated and stored at − 70 °C until batch analysis. Routine biochemical and hematological parameters were measured in a central laboratory using standardized procedures. Lipid profiles, including total cholesterol, triglycerides, high-density lipoprotein cholesterol (HDL-C), low-density lipoprotein cholesterol (LDL-C), apolipoprotein A1 (ApoA1), and apolipoprotein B (ApoB), were determined using validated methods. Total cholesterol and triglycerides were measured by enzymatic assays. HDL-C was measured in the supernatant after magnesium chloride–phosphotungstate precipitation, and LDL-C concentrations were calculated based on the precipitation method. Serum ApoA1 and ApoB concentrations were quantified using turbidimetric immunoassays with commercially available reagents. All lipid and apolipoprotein measurements were performed in batches under routine internal quality-control procedures to minimize inter-assay variability [14].

### Data preprocessing

Among participants recruited from villages 1 to 10, a small proportion of missing data was identified across candidate baseline variables (Supplementary Table S1). Participants were first randomly divided into training and internal validation sets in a 7:3 ratio. Multiple imputation by chained equations was then performed only within the training cohort for variables with missing data prior to feature selection. These variables were considered during the initial feature selection process but were not retained in the final model. Neither the internal validation cohort nor the geographically distinct external validation cohort was involved in the imputation process.

Following imputation, participants were randomly divided into training and internal validation sets in a 7:3 ratio. Categorical variables were encoded as binary indicators. ML models evaluated included Light Gradient Boosting Machine (LightGBM), Random Forest (RF), Logistic Regression (LR), Support Vector Machine (SVM), Multilayer Perceptron (MLP), and K-Nearest Neighbours (KNN). Continuous variables were not standardised for tree-based models and logistic regression (LightGBM, RF, and LR), given their insensitivity to feature scaling, whereas standardisation was applied for SVM, MLP, and KNN to optimise numerical stability and model performance. For these models, scaling parameters were derived from the training cohort and applied unchanged to the internal and external validation cohorts. The data in the external validation cohort (villages 11 to 14) underwent the same preprocessing procedures. A schematic overview of the model development, preprocessing, and validation workflow is provided in Supplementary Figure S1.

### Feature selection

Feature selection was performed in the training dataset to identify the most relevant predictors for sudden cardiac death while reducing model complexity and redundancy. The Boruta algorithm, implemented as a wrapper around random forest classification, was applied in the training dataset to evaluate the relevance of all candidate baseline predictors by comparing their importance with that of permuted shadow variables over iterative resampling [15]. Only predictors classified as confirmed important were retained for subsequent model development. Given the limited sample size, only features classified as confirmed important (green features) were retained for subsequent model development. Subsequently, pairwise Pearson correlation analysis was conducted among the retained features, and variables with high collinearity (|r| > 0.6) were excluded. The remaining features were included for subsequent model development.

### Model development and validation

Model development was performed by incorporating the selected features into the six previously described ML algorithms. All models were trained exclusively on the training cohort, hyperparameters were initially optimised using grid search with five-fold cross-validation, followed by manual fine-tuning to further improve model performance.

To address class imbalance (around 1:42), class weighting was applied in LightGBM, RF, LR, and SVM models using a balanced weighting scheme. For MLP and KNN models, which do not natively support class weighting, the Synthetic Minority Over-sampling Technique (SMOTE) was applied to the training cohort.

Following model training, performance was evaluated in both the internal and external validation cohorts. Discriminative ability was primarily assessed using receiver operating characteristic (ROC) curves, with the area under the curve (AUC) and corresponding 95% confidence intervals (CI) calculated. Additional performance metrics included accuracy, specificity, precision, recall (sensitivity), F1 score, and G-mean. To further evaluate the potential clinical utility of the models, decision curve analysis (DCA) was performed to quantify net benefit across a range of risk thresholds.

For the final selected ML model, calibration was additionally assessed in the internal and external validation cohorts using calibration plots and Brier scores. To assess the robustness of the primary findings to different class imbalance handling strategies, sensitivity analyses were performed by re-training the final model using alternative approaches, including no imbalance correction, SMOTE, and random undersampling.

Additional time-specific outcome analyses were performed by redefining sudden cardiac death as events occurring within 5, 10, and 15 years from baseline, and the performance of the final model was re-evaluated across these alternative time horizons.

To further evaluate the incremental predictive contribution of lipid-related biomarkers, an additional staged model comparison analysis was performed. Specifically, three models were constructed: (1) a clinical model including Age, SBP, CAD, LVH, and Total Ferritin; (2) a model additionally incorporating conventional lipid markers (Triglyceride, HDL-C, and LDL-C); and (3) the final full model further incorporating advanced lipid biomarkers (Apolipoprotein A1 and Apolipoprotein B). Model discrimination was compared using AUC with 95% confidence intervals, and formal pairwise comparisons against the final model were performed using the DeLong test.

### Optimal model selection and interpretation

Based on these evaluation metrics, the optimal model was identified by jointly considering discrimination performance and overall classification metrics. For the selected model, the optimal decision threshold was determined in the internal validation cohort using the Youden index, defined as the threshold maximising the sum of sensitivity and specificity, and was subsequently applied unchanged to the external validation cohort. Positive predictive value (PPV) and negative predictive value (NPV) were subsequently calculated at the optimal threshold. To enhance interpretability, SHapley Additive exPlanations (SHAP) were applied to the final model to quantify feature contributions and to elucidate the direction and magnitude of individual predictors on model predictions.

### Web-based risk calculator

A web-based calculator implementing the optimal model was developed, allowing users to obtain individualised risk estimates through a user-friendly interface.

### Statistical analysis

Continuous variables are presented as mean ± standard deviation or median with interquartile range (IQR), as appropriate, and were compared using the Student’s t test or the Mann–Whitney U test. Categorical variables are expressed as counts and percentages and were compared using the chi-square test or Fisher’s exact test, as appropriate.

All statistical analyses were conducted using Python (version 3.11) and R (version 4.2.1). Machine learning analyses were performed using established Python libraries, including scikit-learn and LightGBM. A two-sided P value < 0.05 was considered statistically significant. To enhance reproducibility, the analysis code used for feature selection, model development, validation, calibration assessment, and sensitivity analyses is provided as Supplementary Code S1.

## Results

### Baseline characteristics

A total of 3,172 participants recruited from 14 villages in the CCCC were included (median [IQR]: 54.9 [45.8, 64.0] years; 1,502 [47.4%] male), among whom 74 experienced SCD during follow-up. Participants who developed SCD were generally older, more frequently male, and had a higher baseline cardiovascular risk profile, as reflected by elevated Framingham and PROCAM risk scores. Differences were also observed in comorbidity burden, ECG parameters, and laboratory profiles, with lower renal function and adverse lipid profiles among individuals who experienced SCD. Detailed baseline characteristics stratified by SCD status are presented in Table 1.

**Table 1:** Baseline characteristics of participants stratified by SCD status

	All	Non-SCD	SCD	*P*
*N*	3,172	3,098	74	
Age, years	54.9 (45.8, 64.0)	54.2 (45.1, 63.9)	65.9 (57.7, 72.3)	< 0.001
Male, n (%)	1,502 (47.4)	1,452 (46.9)	50 (67.6)	< 0.001
Body mass index, kg/m^2^	23.2 (21.2, 25.6)	23.2 (21.2, 25.6)	23.0 (21.1, 25.6)	0.673
Systolic blood pressure, mmHg	120.0 (110.0, 138.0)	120.0 (110.0, 138.0)	129.0 (118.0, 148.0)	0.002
Diastolic blood pressure, mmHg	76.0 (70.0, 84.0)	76.0 (70.0, 84.0)	78.0 (70.0, 86.0)	0.291
Heart rate, beats/min	64.0 (58.0, 72.0)	64.0 (58.0, 72.0)	63.0 (56.0, 69.0)	0.090
Smoking, n (%)	1,173 (37.0)	1,132 (36.5)	41 (55.4)	0.001
Drinking, n (%)	951 (30.0)	926 (29.9)	25 (33.8)	0.521
Framingham risk score	13.0 (9.0, 15.0)	13.0 (9.0, 15.0)	15.0 (13.0, 18.0)	< 0.001
Cardiovascular risk PROCAM score	40.0 (26.0, 51.0)	40.0 (26.0, 51.0)	52.0 (43.0, 60.0)	< 0.001
Comorbidities, n (%)				
Prior atrial fibrillation	38 (1.2)	38 (1.2)	0 (0.0)	0.338
Coronary artery disease	79 (2.5)	54 (1.7)	25 (33.8)	< 0.001
Diabetes mellitus	928 (29.3)	910 (29.4)	18 (24.3)	0.369
Heart failure	26 (0.8)	25 (0.8)	1 (1.4)	0.460
Cerebrovascular accident	144 (4.5)	140 (4.5)	4 (5.4)	0.577
Hypertension	972 (30.6)	934 (30.1)	38 (51.4)	< 0.001
Chronic pulmonary disease	7 (0.2)	7 (0.2)	0 (0.0)	0.682
Medications, n (%)				
Hypertension medicine	378 (11.9)	356 (11.5)	22 (29.7)	< 0.001
Diabetes mellitus medicine	118 (3.7)	115 (3.7)	3 (4.1)	0.878
Electrocardiography parameters, n (%)				
LVH	258 (8.1)	239 (7.7)	19 (25.7)	< 0.001
RVH	8 (0.3)	7 (0.2)	1 (1.4)	0.056
ST change	226 (7.1)	223 (7.2)	3 (4.1)	0.299
Atrial premature contraction	32 (1.0)	27 (0.9)	5 (6.8)	< 0.001
Ventricular premature contraction	57 (1.8)	56 (1.8)	1 (1.4)	0.770
Left bundle branch block	25 (0.8)	24 (0.8)	1 (1.4)	0.579
Right bundle branch block	113 (3.6)	111 (3.6)	2 (2.7)	0.686
Atrial fibrillation / atrial flutter	34 (1.1)	33 (1.1)	1 (1.4)	0.813
Atrioventricular block	81 (2.6)	79 (2.6)	2 (2.7)	0.934
Long QT	154 (4.9)	149 (4.8)	5 (6.8)	0.406
Laboratory results				
Fasting glucose, mg/dL	103.0 (96.0, 113.0)	103.0 (96.0, 113.0)	104.0 (96.0, 115.0)	0.466
White blood cell, ×10^9^/L	6.1 (5.1, 7.2)	6.1 (5.1, 7.2)	6.3 (5.3, 7.8)	0.289
Lymphocyte, %	22.0 (18.0, 26.8)	22.0 (18.0, 26.0)	22.0 (16.0, 28.0)	0.865
Platelet, ×10^9^/L	262.0 (216.0, 313.0)	262.0 (216.0, 314.0)	249.0 (208.0, 296.0)	0.175
Mean corpuscular volume, fL	93.5 (89.9, 96.8)	93.4 (89.9, 96.7)	95.1 (91.3, 98.8)	0.018
Haemoglobin, g/dL	13.8 (12.8, 15.0)	13.8 (12.8, 15.0)	13.9 (12.9, 14.9)	0.857
Albumin, g/L	4.6 (4.4, 4.9)	4.6 (4.4, 4.9)	4.5 (4.2, 4.9)	0.025
Prealbumin, mg/L	26.5 (22.6, 31.2)	26.5 (22.6, 31.2)	26.8 (22.3, 31.7)	0.846
eGFR, mL/min/1.73 m^2^	91.9 (74.3, 106.4)	92.3 (74.9, 106.5)	76.4 (64.5, 93.8)	< 0.001
Blood urea nitrogen, mg/dL	17.0 (14.0, 22.0)	17.0 (14.0, 22.0)	19.0 (16.0, 23.0)	0.001
RDW, %	13.1 (12.7, 13.7)	13.1 (12.7, 13.7)	13.2 (12.7, 13.9)	0.199
Transferrin, mg/dL	271.0 (243.3, 303.0)	272.0 (244.0, 303.0)	258.5 (227.0, 289.0)	0.014
Triglycerides, mg/dL	100.0 (70.0, 150.0)	100.0 (70.0, 150.0)	103.5 (74.0, 161.0)	0.351
Total cholesterol, mg/dL	194.0 (169.0, 225.0)	194.0 (169.0, 225.0)	204.0 (179.0, 244.0)	0.099
HDL-C, mg/dL	46.0 (38.0, 55.0)	46.0 (38.0, 55.0)	43.5 (36.0, 55.0)	0.268
LDL-C, mg/dL	135.0 (109.0, 165.0)	135.0 (109.0, 165.0)	141.5 (115.0, 182.0)	0.078
Apolipoprotein A1, g/L	1.3 (1.1, 1.5)	1.3 (1.1, 1.5)	1.3 (1.1, 1.5)	0.239
Apolipoprotein B, g/L	0.9 (0.7, 1.1)	0.9 (0.7, 1.1)	1.0 (0.8, 1.3)	0.023
Lipoprotein(a), mg/L	92.7 (40.2, 194.5)	92.5 (40.2, 193.1)	111.6 (46.7, 234.0)	0.250

*Abbreviations: eGFR* estimated glomerular filtration rate, *HDL-C* high-density lipoprotein cholesterol, *LDL-C* low-density lipoprotein cholesterol, *LVH* left ventricular hypertrophy, *RDW* red cell distribution width, *RVH* right ventricular hypertrophy, *SCD* sudden cardiac death

### Predictors selected for model development

Using the Boruta feature selection procedure in the training cohort, twelve candidate predictors were initially identified as important for SCD prediction (Supplementary Figure S2). Following further assessment of collinearity, highly correlated variables were excluded (Supplementary Figure S3), resulting in a final set of ten predictors. These included age, systolic blood pressure (SBP), coronary artery disease (CAD), left ventricular hypertrophy (LVH), transferrin, and lipid profile (including triglycerides, HDL-C, LDL-C, ApoA1, and ApoB). The distributions of these selected predictors, together with the proportion of SCD events, across the training, internal validation, and external validation cohorts are presented in Supplementary Table S2. While the training and internal validation cohorts showed broadly similar distributions, some differences were observed in the geographically distinct external validation cohort, including age, SBP, and several lipid-related variables.

### Model performance evaluation and comparison

**Fig. 1:**
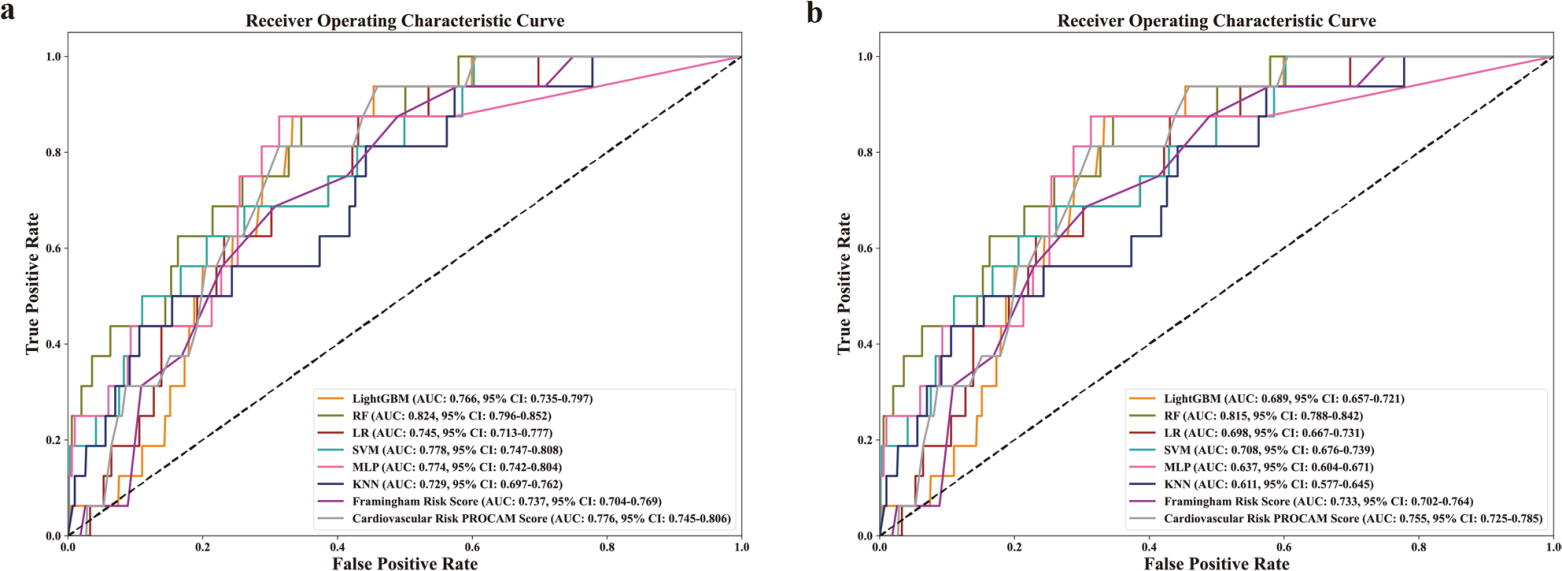
Receiver operating characteristic curves of machine learning models in the internal (**a**) and external (**b**) validation cohorts. AUC, area under the curve; CI, confidence interval; KNN, K-Nearest Neighbours; LightGBM, Light Gradient Boosting Machine; LR, Logistic Regression; MLP, Multilayer Perceptron; RF, Random Forest; SVM, Support Vector Machine

The selected predictors were used to develop the predefined ML models in the training cohort, with final hyperparameter settings summarised in Supplementary Table S3. Discriminative performance was evaluated using ROC analysis (Fig. 1). In the internal validation cohort, the RF achieved the highest AUC (0.824), compared with other ML models (AUCs: 0.729–0.778), as well as the traditional cardiovascular scores (Framingham Risk Score [AUC: 0.737] and the Cardiovascular Risk PROCAM Score [AUC: 0.776]). DeLong test results comparing AUCs with the Framingham Risk Score as the reference (Supplementary Table S4), only the RF showed significantly higher AUCs in both the internal (ΔAUC: 0.087, *P* = 0.027) and external validation cohorts (ΔAUC: 0.082, *P* = 0.008), while no significant differences were observed for the other ML models or the Cardiovascular Risk PROCAM Score.

**Fig. 2:**
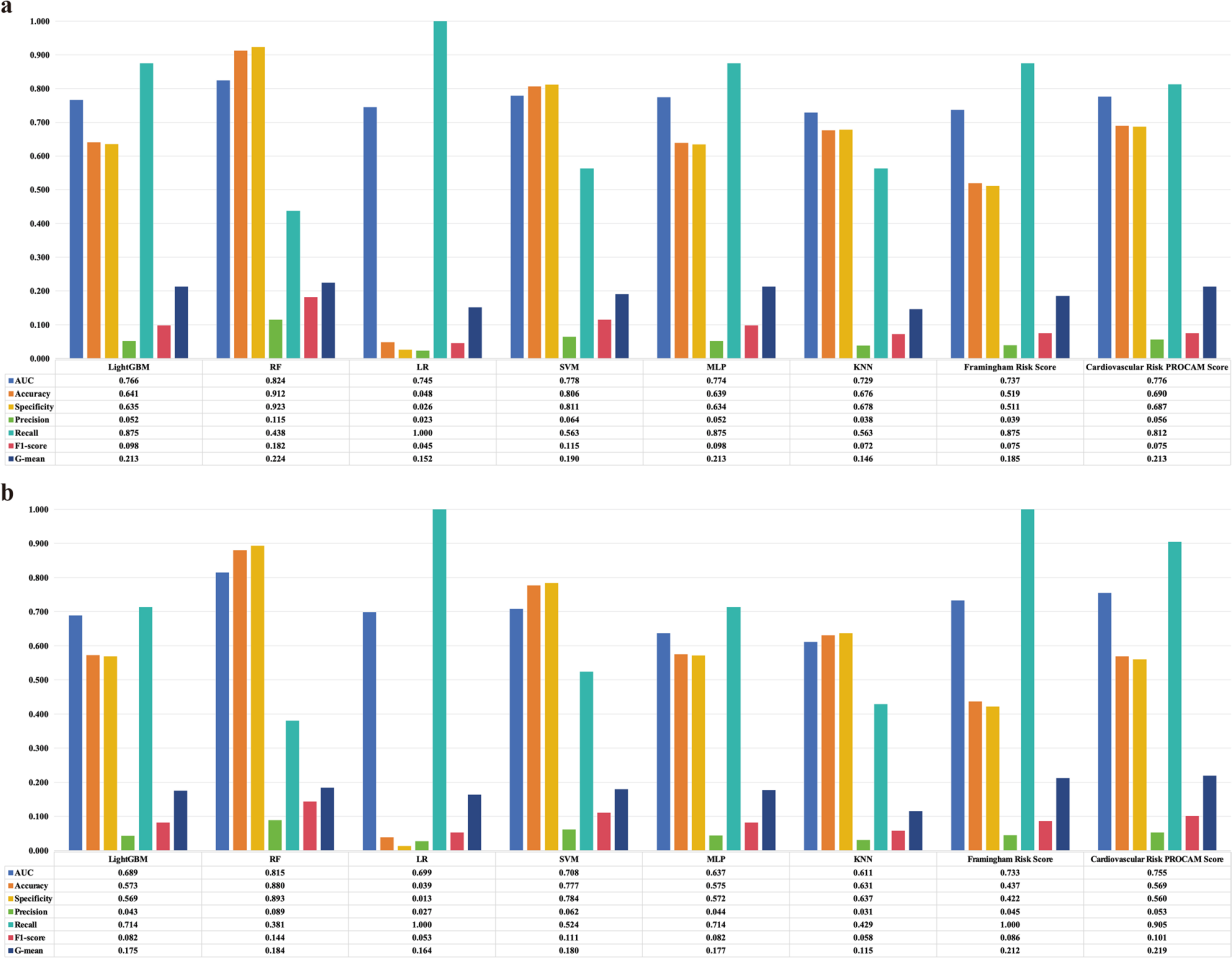
Performance metrics of machine learning models and traditional risk scores in the internal (**a**) and external (**b**) validation cohorts. AUC, area under the curve; KNN, K-Nearest Neighbours; LightGBM, Light Gradient Boosting Machine; LR, Logistic Regression; MLP, Multilayer Perceptron; RF, Random Forest; SVM, Support Vector Machine

**Fig. 3:**
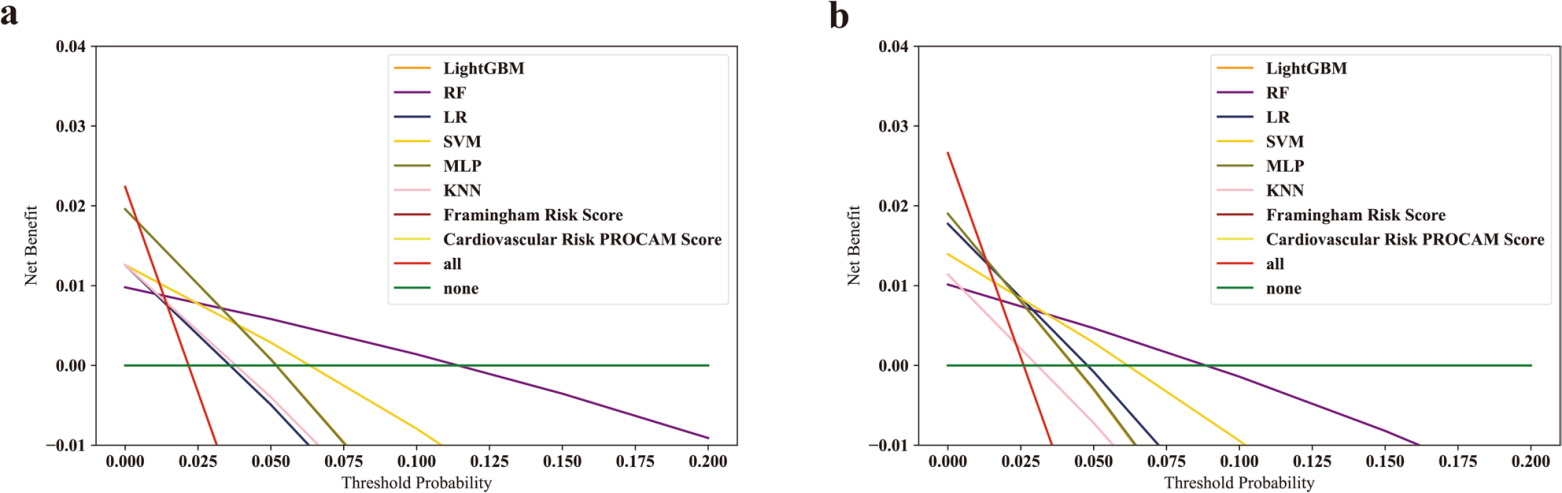
Decision curve analysis of machine learning models and traditional risk scores in the internal (**a**) and external (**b**) validation cohorts. Net benefit is shown across threshold probabilities, with reference strategies of treating all (red) or none (green). KNN, K-Nearest Neighbours; LightGBM, Light Gradient Boosting Machine; LR, Logistic Regression; MLP, Multilayer Perceptron; RF, Random Forest; SVM, Support Vector Machine

Performance metrics across models are shown in Fig. 2 & Supplementary Table S5. In the internal validation cohort, the RF achieved the highest classification accuracy (0.912), together with the highest F1-score (0.182) and G-mean (0.224) among all evaluated models. In the external validation cohort, the RF maintained the highest F1-score (0.144), while its G-mean (0.184) was slightly lower than those observed for the traditional risk scores. Additionally, DCA curve (Fig. 3) demonstrated highest net benefit for the RF model across a range of threshold probabilities compared with other machine learning models and traditional risk scores.

Based on overall AUC, accuracy, and performance in minority class prediction, assessed by F1-score and G-mean in both validation cohorts, the RF was selected as the optimal model.

### Sensitivity analyses and calibration

Calibration analysis of the final RF model was performed in both validation cohorts (Supplementary Figure S4), with Brier scores of 0.0211 and 0.0249 in the internal and external validation cohorts, respectively. Sensitivity analyses using alternative class imbalance handling strategies yielded broadly consistent performance (Supplementary Table S6), supporting the robustness of the primary findings.

Sensitivity analyses using time-specific SCD outcomes at 5, 10, and 15 years showed broadly consistent predictive performance of the RF model across different follow-up horizons (Supplementary Table S7).

Incremental model comparison showed that adding conventional lipid markers to the clinical model yielded modest improvement in internal validation but not in external validation (Supplementary Table S8). In contrast, the full model incorporating advanced lipid biomarkers (ApoA1 and ApoB) achieved the highest discrimination in both cohorts, with significant improvement over reduced models in DeLong comparisons.

### Model interpretability and clinical applicability

**Fig. 4:**
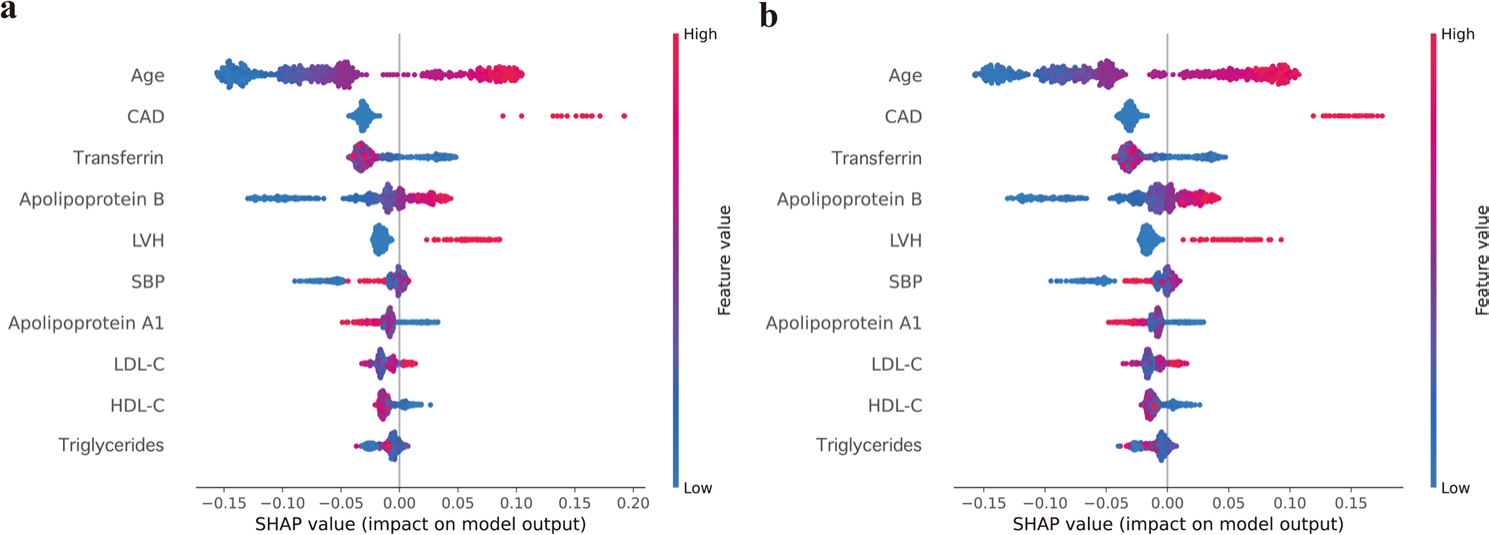
SHapley Additive exPlanations summary plots for the Random Forest model in the internal (**a**) and external (**b**) validation cohorts. Plots show feature importance and direction of effect on model predictions, with red indicating higher feature values and blue indicating lower feature values. CAD, coronary artery disease; HDL-C, high-density lipoprotein cholesterol; LDL-C, low-density lipoprotein cholesterol; LVH, left ventricular hypertrophy; SBP, systolic blood pressure

SHAP summary plots for the random forest model in the internal and external validation cohorts (Fig. 4) showed consistent feature importance patterns across cohorts, with age ranking highest, followed by CAD, transferrin, ApoB, and LVH as the top five contributors.

An optimal probability threshold for the RF was determined using the Youden index (Supplementary Table S9). At this threshold, the model achieved a PPV of 5.5% and a NPV of 99.6% in the internal validation cohort. When applied to the external validation cohort using the same threshold, the model yielded a PPV of 5.4% and an NPV of 99.3%.

**Fig. 5:**
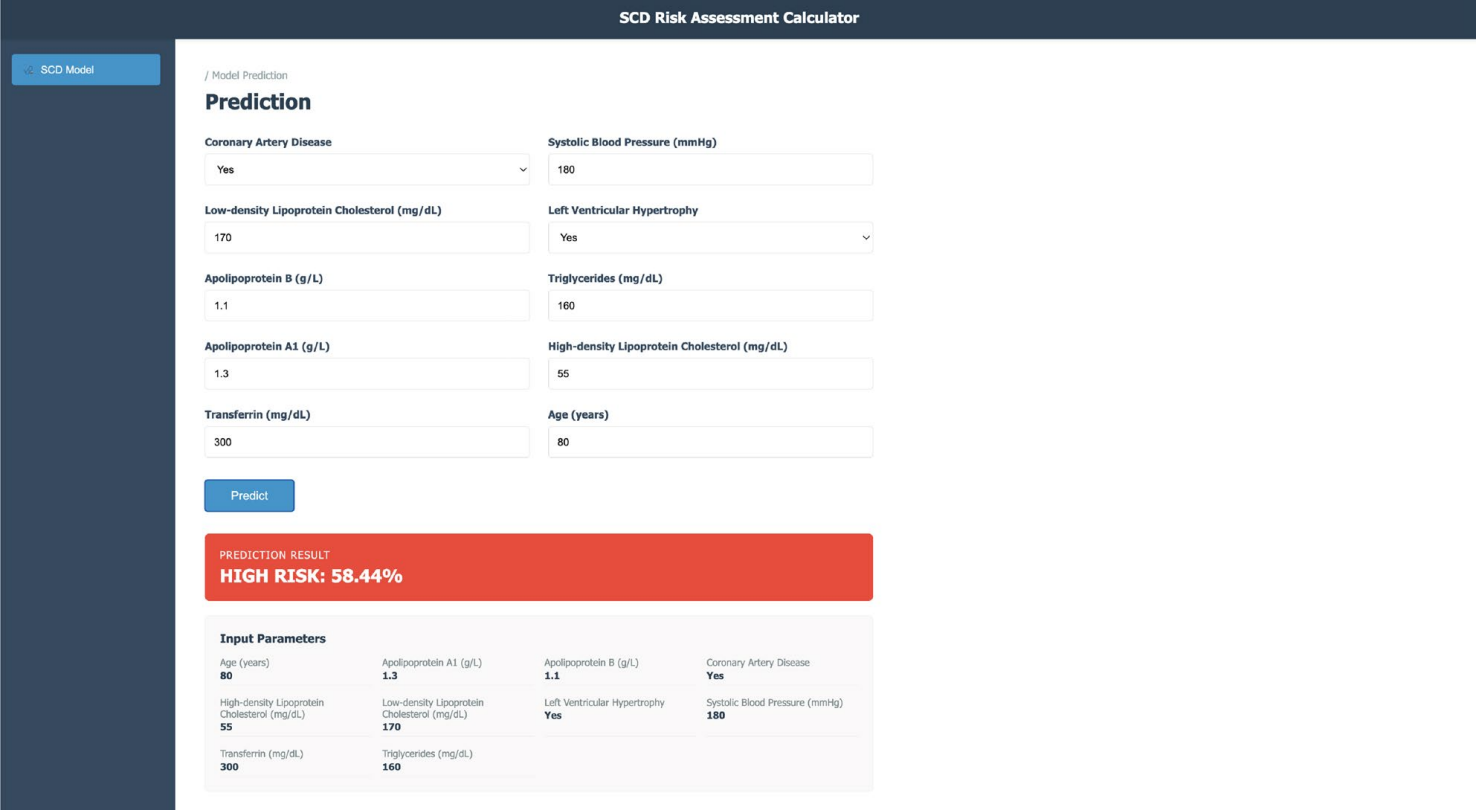
Web-based risk calculator interface for individualised risk of sudden cardiac death estimation based on the random forest model

A web-based risk calculator based on the RF model was developed and is publicly available online at https://qinkaiyu.shinyapps.io/scd-risk-calculator/, allowing users to input the required predictors and obtain an individualised estimated probability of SCD, the user interface is shown in Fig. 5.

## Discussion

In this community-based cohort study, we developed and validated ML models for SCD risk prediction, with the RF demonstrating the most favourable overall performance across internal and external validation cohorts. Importantly, model performance was further evaluated in a geographically distinct validation cohort drawn from separate villages within the same community, providing an assessment of internal geographic robustness rather than true external validation across independent populations. Notably, the RF integrated demographic factors, cardiovascular structural markers, and a detailed lipid profile, with multiple lipid components contributing to risk estimation, underscoring the relevance of multiple lipid measures in long-term SCD risk assessment. Beyond its discriminative ability, a key finding of this study is the consistently high NPV achieved by the RF, the model yielded NPVs exceeding 99% in both validation cohorts, indicating a strong ability to reliably identify individuals at low risk of SCD. In the context of a rare but devastating outcome such as SCD, this high NPV highlights the potential clinical utility of the model as an effective rule-out tool, enabling reassurance and risk stratification in population-based or primary prevention settings while minimizing unnecessary downstream investigations in low-risk individuals.

Despite growing interest in ML for cardiovascular risk stratification, evidence for long-term SCD prediction remains limited. How does this work compare with prior studies using ML approaches for SCD prediction? A recent community-based study using electronic health record data have demonstrated the potential of ML to improve long-term SCD risk prediction, with a RF model incorporating demographic characteristics, comorbidities, vital signs, and routine metabolic markers achieving a test-set AUC of 0.757 [16]. Despite incorporating predictors similar to those in our RF model, including age, SBP, and triglycerides, prior study focused mainly on discrimination metrics, and the lack of reported PPV or NPV limits assessment of their clinical rule-in or rule-out utility. Similarly, large-scale EHR-based artificial intelligence models have demonstrated the feasibility of identifying individuals at elevated SCD risk in the general population, although these approaches relied on highly granular longitudinal diagnostic and prescription data [17]. In addition, ML approaches in selected high-risk populations, such as patients with implantable cardioverter-defibrillators, have shown promising predictive performance using clinical and device-derived variables, although these models targeted arrhythmic surrogate outcomes rather than prospective SCD occurrence [18]. Another ML approach reported high discriminative performance for SCD-related outcomes, based on detailed cardiac morphological features, with external validation AUCs exceeding 0.80 [19]. However, this analysis was largely cross-sectional and diagnostic in nature, relying on post-mortem data, which constrains its applicability to prospective risk stratification in community settings.

Beyond age, SBP, and CAD, which are widely recognized risk factors, the predictors identified by the RF model are biologically plausible and consistent with established mechanisms underlying SCD. LVH represents a key structural substrate for malignant ventricular arrhythmias, reflecting adverse myocardial remodeling and increased electrical instability that may predispose to SCD [20]. Transferrin, a marker of iron metabolism, may reflect systemic inflammatory and oxidative stress states, which have been implicated in myocardial vulnerability and arrhythmogenesis [21, 22], thereby potentially contributing to SCD risk. The inclusion of multiple lipid-related measures in the RF model further emphasizes the contribution of dyslipidemia to SCD risk. Elevated triglycerides and LDL-C and reduced HDL-C are well-established contributors to atherosclerotic burden and cardiovascular risk [23]. Beyond these conventional lipid indices, ApoA1 and ApoB provide additional information on lipoprotein particle composition and atherogenic balance. Apolipoprotein B reflects the total burden of atherogenic particles [24], whereas apolipoprotein A1 is central to reverse cholesterol transport and anti-inflammatory pathways [25]. Together, these measures capture complementary aspects of lipid metabolism and atherogenic risk not reflected by conventional lipid assessment. Incremental model comparison further showed that, although conventional lipid markers alone did not consistently improve performance, the full model incorporating advanced lipid biomarkers (ApoA1 and ApoB) achieved superior discrimination across validation cohorts. This supports the added prognostic value of broader lipid profiling beyond conventional clinical and lipid risk markers.

Not all established SCD risk factors, such as heart failure and prolonged QT interval [26, 27], were retained in the final model. This likely reflects limited incremental predictive contribution within the specific modelling framework rather than lack of clinical relevance. In this community-based cohort with relatively few SCD events, statistical power to detect weaker independent associations may also have been limited. Additionally, baseline treatment or closer clinical surveillance among patients with recognised cardiovascular conditions may have attenuated the predictive contribution of some traditional risk markers.

From a clinical perspective, the consistently high negative predictive value observed for the RF is particularly relevant. Given the low incidence of SCD in this cohort, the modest PPV was expected, suggesting that the model may be more suitable for risk stratification or rule-out assessment rather than as a stand-alone rule-in tool. SCD is a relatively rare but catastrophic outcome, and in such low-incidence settings, models designed for population-level screening inevitably face limitations in positive predictive performance. In this context, the ability to reliably identify individuals at low risk assumes greater practical importance. The high NPV achieved by the model supports its potential role as a rule-out tool, allowing reassurance of low-risk individuals and more efficient allocation of preventive resources. In practice, the model may be used as an initial community-based screening or risk stratification tool to identify individuals unlikely to require further cardiovascular evaluation, rather than as a stand-alone tool for selecting high-risk patients for invasive preventive interventions. Such an approach may be especially valuable in community-based or primary prevention settings, where minimizing unnecessary downstream investigations while maintaining patient safety is a key consideration.

Further validation in larger, independent, and prospective cohorts across different geographic regions and ethnic populations is required to confirm model robustness and generalizability. Accordingly, the current model should be considered exploratory and hypothesis-generating rather than ready for broad clinical implementation. Additionally, prospective studies evaluating the integration of the model into clinical workflows are needed to determine its real-world impact on clinical decision-making and outcomes. The availability of an online risk calculator based on the final model represents an initial step toward clinical translation, providing a scalable platform for population-level risk assessment and facilitating future implementation studies. If prospectively validated, such a tool could potentially be integrated into community screening programmes or primary care workflows as a decision-support aid for preliminary risk assessment.

### Limitations

Several limitations should be considered. First, despite the prospective design and long-term follow-up, the number of SCD events was relatively small, reflecting the low incidence of SCD in community-based populations. This limited event count increases the risk of model overfitting and may reduce the stability of performance estimates, despite the use of internal and geographically partitioned external validation, class imbalance handling strategies, and sensitivity analyses. This class imbalance may limit positive predictive performance, and the primary clinical value of the model lies in its high negative predictive capability for risk exclusion. Second, although validation was performed using geographically distinct villages, all participants were derived from a single regional cohort in Taiwan with relatively homogeneous ethnic and environmental characteristics. Therefore, the findings should not be interpreted as evidence of broad external generalisability, and validation in independent populations is required. Third, predictor variables were obtained at baseline only, and temporal changes in risk factors and treatments were not incorporated, although dynamic changes in lipid biomarker may provide additional predictive value for long-term risk estimation [28]. This may limit the model’s ability to capture evolving risk profiles over time. Fourth, changes in clinical management and risk factor control over time were not accounted for and may have had a meaningful impact on long-term SCD risk estimation. Additionally, the current classification-based modelling framework treated SCD as a binary outcome and did not explicitly account for competing risks from non-SCD mortality, which may influence long-term risk estimation in an ageing population. Finally, the model was designed to estimate long-term SCD risk and does not provide information on short-term or imminent risk, which may be relevant in certain clinical contexts. Further validation in larger and truly independent prospective cohorts across different geographic regions and ethnic populations will be required to confirm its reliability and support broader clinical application.

## Conclusion

Our study developed and geographically validated an RF-based model incorporating demographic factors, cardiovascular structural markers, and multiple lipid profile components for long-term SCD risk prediction in a Taiwanese community-based population, with implementation as an online risk calculator. The model demonstrated robust discrimination and consistently high negative predictive values across internal and independent external validation cohorts, supporting its potential utility as a rule-out tool for identifying individuals at low risk of SCD, pending further validation in independent populations.

## Supplementary Information

Supplementary Material 1.

## Data Availability

The data used in this study are derived from the Chin-Shan Community Cardiovascular Cohort (CCCC). Access to the dataset is restricted and not publicly available due to privacy regulations and institutional policies. However, data may be available upon reasonable request and with appropriate ethical approval. The dataset is under the custodianship of Yun-Yu Chen, MPH, PhD, and Kuo-Liong Chien, MD, PhD. Requests for data access should be directed to the corresponding authors.

## Abbreviations

ApoA1: Apolipoprotein A1
ApoB: Apolipoprotein B
AUC: Area Under the Curve
CAD: Coronary Artery Disease
CCCC: Chin-Shan Community Cardiovascular Cohort
CI: Confidence Interval
DCA: Decision Curve Analysis
ECG: Electrocardiogram
F1-score: Harmonic Mean of Precision and Recall
HDL-C: High-Density Lipoprotein Cholesterol
IQR: Interquartile Range
KNN: K-Nearest Neighbours
LDL-C: Low-Density Lipoprotein Cholesterol
LightGBM: Light Gradient Boosting Machine
LR: Logistic Regression
LVH: Left Ventricular Hypertrophy
ML: Machine Learning
MLP: Multilayer Perceptron
NPV: Negative Predictive Value
PPV: Positive Predictive Value
PROCAM: Prospective Cardiovascular Münster Study
RF: Random Forest
ROC: Receiver Operating Characteristic
SBP: Systolic Blood Pressure
SCD: Sudden Cardiac Death
SHAP: SHapley Additive exPlanations
SVM: Support Vector Machine
