# Supplementary material for: Machine learning prediction of sudden cardiac death incorporating multiple lipid markers: evidence from the Taiwan Chin Shan community cohort
Source: Lipids Health Dis. 2026 May 30;25:171. doi: 10.1186/s12944-026-02984-5 (PMC13430708; doi:10.1186/s12944-026-02984-5)
Supplement: Supplementary file 1 — Supplementary Material 1. [file 12944_2026_2984_MOESM1_ESM.docx]

Supplementary Code S1. Python code for machine learning model development, validation, calibration, and sensitivity analyses for sudden cardiac death prediction

# =========================

# Basic libraries

# =========================

import warnings

warnings.filterwarnings("ignore")

import random

from collections import Counter

import numpy as np

import pandas as pd

# =========================

# Plotting

# =========================

import matplotlib.pyplot as plt

import matplotlib as mpl

import seaborn as sns

# =========================

# Statistical analysis

# =========================

from scipy import stats

from scipy.stats import randint, uniform

import statsmodels.api as sm

# =========================

# Data preprocessing

# =========================

from sklearn.model_selection import train_test_split, GridSearchCV, RandomizedSearchCV

from sklearn.preprocessing import StandardScaler, MinMaxScaler, label_binarize

from imblearn.over_sampling import SMOTE

# =========================

# Machine learning models

# =========================

import lightgbm as lgb

from sklearn.linear_model import LogisticRegression, LinearRegression

from sklearn.ensemble import RandomForestClassifier

from sklearn.svm import SVC

from sklearn.neighbors import KNeighborsClassifier

from sklearn.neural_network import MLPClassifier

from sklearn.multiclass import OneVsRestClassifier

# =========================

# Feature selection

# =========================

from sklearn.feature_selection import RFE

from sklearn.linear_model import RidgeCV, LassoCV, Ridge, Lasso

# =========================

# Model evaluation

# =========================

from sklearn.metrics import (

roc_auc_score,

roc_curve,

auc,

accuracy_score,

precision_score,

recall_score,

f1_score,

average_precision_score,

precision_recall_curve,

confusion_matrix,

classification_report

)

from sklearn.calibration import calibration_curve, CalibratedClassifierCV

# =========================

# Explainability

# =========================

import shap

# =========================

# Candidate features

# =========================

target = "scd"

feature_list = [

"age", "male", "bmi", "sbp", "dbp", "hr",

"smoke_gr", "drink_gr",

"AF_hx", "cad0", "HF", "cva0", "dm", "ht0", "PULM_DZ",

"ht_med", "dm_med",

"LVH", "RVH", "ANY_ST", "QT", "APC", "VPC", "AV",

"AFIB_LUT", "LBBB", "RBBB",

"GLU", "CHOL", "TRIG", "HDL", "LDL",

"LIPOA", "APOA1", "APOB",

"T_FERRIN", "S_WBC", "PREALB", "RDW_CV", "PLT",

"WBC", "MCV", "HGB", "BUN", "GFR", "ALB"

]

# =========================

# Development/internal split

# =========================

y = df[target].astype(int)

X = df[feature_list]

X_train, X_test, y_train, y_test = train_test_split(

X,

y,

test_size=0.3,

random_state=167,

stratify=y

)

print("Train shape:", X_train.shape, y_train.shape)

print("Test shape :", X_test.shape, y_test.shape)

# =========================

# Save split datasets

# =========================

train_df = X_train.copy()

train_df[target] = y_train.values

train_df["CASE"] = df.loc[X_train.index, "CASE"]

train_df["fram_score"] = df.loc[X_train.index, "fram_score"]

train_df["procam_score"] = df.loc[X_train.index, "procam_score"]

test_df = X_test.copy()

test_df[target] = y_test.values

test_df["CASE"] = df.loc[X_test.index, "CASE"]

test_df["fram_score"] = df.loc[X_test.index, "fram_score"]

test_df["procam_score"] = df.loc[X_test.index, "procam_score"]

train_df.to_csv("train.csv", index=False)

test_df.to_csv("test.csv", index=False)

# =========================

# External validation dataset

# =========================

ext_df = dt.copy()

# =========================

# Final selected features

# =========================

final_features = [

"CAD", "SBP", "LDL-C", "LVH", "Apolipoprotein B",

"Triglyceride", "Apolipoprotein A1", "HDL-C",

"Total Ferritin", "Age"

]

target = "scd"

# Training set

X_train = train_df[final_features].copy()

y_train = train_df[target].astype(int)

# Internal validation set

X_test = test_df[final_features].copy()

y_test = test_df[target].astype(int)

# External validation set

X_ex = ext_df[final_features].copy()

y_ex = ext_df[target].astype(int)

print("X_train:", X_train.shape, "y_train:", y_train.shape)

print("X_test :", X_test.shape, "y_test :", y_test.shape)

print("X_ext :", X_ex.shape, "y_ext :", y_ex.shape)

# =========================

# Boruta feature selection

# =========================

from boruta import BorutaPy

from sklearn.ensemble import RandomForestClassifier

import pandas as pd

import numpy as np

# Random forest estimator for Boruta

rf_boruta = RandomForestClassifier(

n_estimators=1000,

random_state=32,

class_weight="balanced",

max_depth=5,

n_jobs=-1

)

# Boruta feature selection

boruta_selector = BorutaPy(

estimator=rf_boruta,

n_estimators="auto",

max_iter=100,

alpha=0.05,

random_state=32,

verbose=2

)

boruta_selector.fit(

X_train.values,

y_train.values

)

# Selected features

boruta_results = pd.DataFrame({

"Feature": X_train.columns,

"Boruta_support": boruta_selector.support_,

"Boruta_weak_support": boruta_selector.support_weak_,

"Boruta_ranking": boruta_selector.ranking_

})

boruta_results = boruta_results.sort_values(

by=["Boruta_ranking", "Feature"]

)

print(boruta_results)

selected_features_boruta = boruta_results.loc[

boruta_results["Boruta_support"] == True,

"Feature"

].tolist()

print("Selected features by Boruta:")

print(selected_features_boruta)

boruta_results.to_csv(

"boruta_feature_selection_results.csv",

index=False

)

# =========================

# Randomized hyperparameter tuning template

# =========================

from sklearn.model_selection import RandomizedSearchCV

param_dist = {

"parameter_1": [...],

"parameter_2": [...],

"parameter_3": [...]

}

model = YourModelClass()

random_search = RandomizedSearchCV(

estimator=model,

param_distributions=param_dist,

n_iter=50,

cv=5,

scoring="roc_auc",

n_jobs=-1,

random_state=42,

verbose=1

)

random_search.fit(X_train, y_train)

print("Best parameters:", random_search.best_params_)

best_model = random_search.best_estimator_

# =========================

# Model training and evaluation

# =========================

# ---------- Random Forest ----------

model_rf = RandomForestClassifier(

n_estimators=516,

random_state=32,

class_weight="balanced",

max_depth=3,

min_samples_leaf=4,

min_samples_split=2,

criterion="gini"

)

model_rf.fit(X_train, y_train)

y_pred_prob_rf = model_rf.predict_proba(X_test)[:, 1]

y_pred_prob_rf_ex = model_rf.predict_proba(X_ex)[:, 1]

print("RF internal AUC:", roc_auc_score(y_test, y_pred_prob_rf))

print("RF external AUC:", roc_auc_score(y_ex, y_pred_prob_rf_ex))

# ---------- Logistic Regression ----------

model_lr = LogisticRegression(

C=0.5,

penalty="l2",

solver="sag",

max_iter=200,

random_state=5,

class_weight="balanced"

)

model_lr.fit(X_train, y_train)

y_pred_prob_lr = model_lr.predict_proba(X_test)[:, 1]

y_pred_prob_lr_ex = model_lr.predict_proba(X_ex)[:, 1]

print("LR internal AUC:", roc_auc_score(y_test, y_pred_prob_lr))

print("LR external AUC:", roc_auc_score(y_ex, y_pred_prob_lr_ex))

# ---------- LightGBM ----------

model_lgb = lgb.LGBMClassifier(

boosting_type="gbdt",

objective="binary",

colsample_bytree=0.8,

learning_rate=0.005,

max_depth=5,

min_child_samples=195,

n_estimators=54,

num_leaves=31,

subsample=0.9,

class_weight="balanced"

)

model_lgb.fit(X_train, y_train)

y_pred_prob_lgb = model_lgb.predict_proba(X_test)[:, 1]

y_pred_prob_lgb_ex = model_lgb.predict_proba(X_ex)[:, 1]

print("LightGBM internal AUC:", roc_auc_score(y_test, y_pred_prob_lgb))

print("LightGBM external AUC:", roc_auc_score(y_ex, y_pred_prob_lgb_ex))

# ---------- Support Vector Machine ----------

model_svm = SVC(

C=0.4,

kernel="rbf",

gamma="auto",

tol=0.0001,

probability=True,

class_weight="balanced"

)

model_svm.fit(X_train, y_train)

y_pred_prob_svm = model_svm.predict_proba(X_test)[:, 1]

y_pred_prob_svm_ex = model_svm.predict_proba(X_ex)[:, 1]

print("SVM internal AUC:", roc_auc_score(y_test, y_pred_prob_svm))

print("SVM external AUC:", roc_auc_score(y_ex, y_pred_prob_svm_ex))

# ---------- SMOTE for KNN and MLP ----------

smote = SMOTE(random_state=42)

X_train_smote, y_train_smote = smote.fit_resample(X_train, y_train)

# ---------- K-Nearest Neighbours ----------

model_knn = KNeighborsClassifier(

n_neighbors=25,

weights="distance",

metric="chebyshev",

p=5

)

model_knn.fit(X_train_smote, y_train_smote)

y_pred_prob_knn = model_knn.predict_proba(X_test)[:, 1]

y_pred_prob_knn_ex = model_knn.predict_proba(X_ex)[:, 1]

print("KNN internal AUC:", roc_auc_score(y_test, y_pred_prob_knn))

print("KNN external AUC:", roc_auc_score(y_ex, y_pred_prob_knn_ex))

# ---------- Multilayer Perceptron ----------

model_mlp = MLPClassifier(

solver="adam",

activation="relu",

hidden_layer_sizes=(2, 4),

alpha=0.0001,

max_iter=500,

random_state=3

)

model_mlp.fit(X_train_smote, y_train_smote)

y_pred_prob_mlp = model_mlp.predict_proba(X_test)[:, 1]

y_pred_prob_mlp_ex = model_mlp.predict_proba(X_ex)[:, 1]

print("MLP internal AUC:", roc_auc_score(y_test, y_pred_prob_mlp))

print("MLP external AUC:", roc_auc_score(y_ex, y_pred_prob_mlp_ex))

# =========================

# ROC curves for all ML models and conventional scores

# =========================

import matplotlib.pyplot as plt

from sklearn.metrics import roc_curve, roc_auc_score

# Editable PDF settings

plt.rcParams["pdf.fonttype"] = 42

plt.rcParams["ps.fonttype"] = 42

plt.rcParams["font.family"] = "Arial"

plt.rcParams["font.size"] = 11

# =========================

# Predicted probabilities

# =========================

prob_internal = {

"RF": model_rf.predict_proba(X_test)[:, 1],

"LightGBM": model_lgb.predict_proba(X_test)[:, 1],

"LR": model_lr.predict_proba(X_test)[:, 1],

"SVM": model_svm.predict_proba(X_test)[:, 1],

"KNN": model_knn.predict_proba(X_test)[:, 1],

"MLP": model_mlp.predict_proba(X_test)[:, 1],

"Framingham score": test_df["fram_score"],

"PROCAM score": test_df["procam_score"]

}

prob_external = {

"RF": model_rf.predict_proba(X_ex)[:, 1],

"LightGBM": model_lgb.predict_proba(X_ex)[:, 1],

"LR": model_lr.predict_proba(X_ex)[:, 1],

"SVM": model_svm.predict_proba(X_ex)[:, 1],

"KNN": model_knn.predict_proba(X_ex)[:, 1],

"MLP": model_mlp.predict_proba(X_ex)[:, 1],

"Framingham score": ext_df["fram_score"],

"PROCAM score": ext_df["procam_score"]

}

# =========================

# ROC plotting function

# =========================

def plot_all_roc(y_true, prob_dict, title, filename):

fig, ax = plt.subplots(figsize=(6, 6))

for model_name, y_prob in prob_dict.items():

fpr, tpr, _ = roc_curve(y_true, y_prob)

auc_value = roc_auc_score(y_true, y_prob)

ax.plot(

fpr,

tpr,

linewidth=1.8,

label=f"{model_name}, AUC = {auc_value:.3f}"

)

ax.plot(

[0, 1],

[0, 1],

linestyle="--",

linewidth=1.2,

label="Reference"

)

ax.set_xlabel("1 - Specificity")

ax.set_ylabel("Sensitivity")

ax.set_title(title)

ax.legend(frameon=False, fontsize=8, loc="lower right")

ax.set_xlim(0, 1)

ax.set_ylim(0, 1)

fig.tight_layout()

fig.savefig(

filename,

format="pdf",

dpi=300,

bbox_inches="tight"

)

plt.show()

# =========================

# Internal validation ROC

# =========================

plot_all_roc(

y_true=y_test,

prob_dict=prob_internal,

title="Internal Validation",

filename="ROC_all_models_internal.pdf"

)

# =========================

# External validation ROC

# =========================

plot_all_roc(

y_true=y_ex,

prob_dict=prob_external,

title="External Validation",

filename="ROC_all_models_external.pdf"

)

# =========================

# Decision curve analysis

# =========================

import numpy as np

import pandas as pd

import matplotlib.pyplot as plt

plt.rcParams["pdf.fonttype"] = 42

plt.rcParams["ps.fonttype"] = 42

plt.rcParams["font.family"] = "Arial"

plt.rcParams["font.size"] = 11

def calculate_net_benefit(y_true, y_prob, thresholds):

y_true = np.asarray(y_true)

y_prob = np.asarray(y_prob)

n = len(y_true)

net_benefits = []

for pt in thresholds:

y_pred = (y_prob >= pt).astype(int)

tp = np.sum((y_pred == 1) & (y_true == 1))

fp = np.sum((y_pred == 1) & (y_true == 0))

nb = (tp / n) - (fp / n) * (pt / (1 - pt))

net_benefits.append(nb)

return np.array(net_benefits)

def plot_dca(y_true, prob_dict, title, filename):

thresholds = np.linspace(0.01, 0.50, 100)

fig, ax = plt.subplots(figsize=(6, 5))

for model_name, y_prob in prob_dict.items():

nb = calculate_net_benefit(y_true, y_prob, thresholds)

ax.plot(thresholds, nb, linewidth=1.8, label=model_name)

prevalence = np.mean(y_true)

treat_all = prevalence - (1 - prevalence) * thresholds / (1 - thresholds)

treat_none = np.zeros_like(thresholds)

ax.plot(thresholds, treat_all, linestyle="--", linewidth=1.2, label="Treat all")

ax.plot(thresholds, treat_none, linestyle=":", linewidth=1.2, label="Treat none")

ax.set_xlabel("Threshold probability")

ax.set_ylabel("Net benefit")

ax.set_title(title)

ax.legend(frameon=False, fontsize=8)

fig.tight_layout()

fig.savefig(filename, format="pdf", dpi=300, bbox_inches="tight")

plt.show()

prob_internal = {

"RF": model_rf.predict_proba(X_test)[:, 1],

"LightGBM": model_lgb.predict_proba(X_test)[:, 1],

"LR": model_lr.predict_proba(X_test)[:, 1],

"SVM": model_svm.predict_proba(X_test)[:, 1],

"KNN": model_knn.predict_proba(X_test)[:, 1],

"MLP": model_mlp.predict_proba(X_test)[:, 1],

"Framingham score": test_df["fram_score"],

"PROCAM score": test_df["procam_score"]

}

prob_external = {

"RF": model_rf.predict_proba(X_ex)[:, 1],

"LightGBM": model_lgb.predict_proba(X_ex)[:, 1],

"LR": model_lr.predict_proba(X_ex)[:, 1],

"SVM": model_svm.predict_proba(X_ex)[:, 1],

"KNN": model_knn.predict_proba(X_ex)[:, 1],

"MLP": model_mlp.predict_proba(X_ex)[:, 1],

"Framingham score": ext_df["fram_score"],

"PROCAM score": ext_df["procam_score"]

}

plot_dca(

y_true=y_test,

prob_dict=prob_internal,

title="DCA: Internal Validation",

filename="DCA_internal.pdf"

)

plot_dca(

y_true=y_ex,

prob_dict=prob_external,

title="DCA: External Validation",

filename="DCA_external.pdf"

)

# =========================

# DeLong test

# =========================

import numpy as np

from scipy import stats

from sklearn.metrics import roc_auc_score

def compute_midrank(x):

J = np.argsort(x)

Z = x[J]

N = len(x)

T = np.zeros(N, dtype=float)

i = 0

while i < N:

j = i

while j < N and Z[j] == Z[i]:

j += 1

T[i:j] = 0.5 * (i + j - 1) + 1

i = j

T2 = np.empty(N, dtype=float)

T2[J] = T

return T2

def fast_delong(predictions_sorted_transposed, label_1_count):

m = label_1_count

n = predictions_sorted_transposed.shape[1] - m

k = predictions_sorted_transposed.shape[0]

positive_examples = predictions_sorted_transposed[:, :m]

negative_examples = predictions_sorted_transposed[:, m:]

tx = np.empty((k, m))

ty = np.empty((k, n))

tz = np.empty((k, m + n))

for r in range(k):

tx[r, :] = compute_midrank(positive_examples[r, :])

ty[r, :] = compute_midrank(negative_examples[r, :])

tz[r, :] = compute_midrank(predictions_sorted_transposed[r, :])

aucs = tz[:, :m].sum(axis=1) / m / n - (m + 1.0) / (2.0 * n)

v01 = (tz[:, :m] - tx) / n

v10 = 1.0 - (tz[:, m:] - ty) / m

sx = np.cov(v01)

sy = np.cov(v10)

delong_cov = sx / m + sy / n

return aucs, delong_cov

def calc_pvalue(aucs, sigma):

diff = np.array([[1, -1]])

z = np.abs(np.diff(aucs)) / np.sqrt(np.dot(np.dot(diff, sigma), diff.T))

pvalue = 2 * (1 - stats.norm.cdf(z))

return float(pvalue)

def delong_roc_test(y_true, pred_1, pred_2):

y_true = np.asarray(y_true)

pred_1 = np.asarray(pred_1)

pred_2 = np.asarray(pred_2)

order = np.argsort(-y_true)

label_1_count = int(np.sum(y_true))

predictions_sorted = np.vstack((pred_1, pred_2))[:, order]

aucs, delong_cov = fast_delong(predictions_sorted, label_1_count)

pvalue = calc_pvalue(aucs, delong_cov)

return aucs[0], aucs[1], pvalue

def delong_compare_rf(y_true, prob_dict, cohort_name):

rf_prob = prob_dict["RF"]

results = []

for model_name, y_prob in prob_dict.items():

if model_name == "RF":

continue

auc_rf, auc_other, p_value = delong_roc_test(

y_true,

rf_prob,

y_prob

)

results.append({

"Cohort": cohort_name,

"Comparison": f"RF vs {model_name}",

"RF AUC": auc_rf,

"Comparator AUC": auc_other,

"P value": p_value

})

return pd.DataFrame(results)

delong_internal = delong_compare_rf(

y_true=y_test,

prob_dict=prob_internal,

cohort_name="Internal validation"

)

delong_external = delong_compare_rf(

y_true=y_ex,

prob_dict=prob_external,

cohort_name="External validation"

)

delong_results = pd.concat(

[delong_internal, delong_external],

ignore_index=True

)

print(delong_results)

delong_results.to_csv(

"delong_comparison_results.csv",

index=False

)

# =========================

# RF sensitivity analyses for class imbalance handling

# =========================

from sklearn.ensemble import RandomForestClassifier

from sklearn.metrics import roc_auc_score, brier_score_loss

from sklearn.calibration import CalibratedClassifierCV

from imblearn.over_sampling import SMOTE

from imblearn.under_sampling import RandomUnderSampler

from imblearn.pipeline import Pipeline as ImbPipeline

from scipy import stats

import pandas as pd

import numpy as np

def auc_ci_normal(auc_value, n):

z = stats.norm.ppf(0.975)

interval = z * np.sqrt(auc_value * (1 - auc_value) / n)

return auc_value - interval, auc_value + interval

def evaluate_rf_sensitivity(model_name, model):

model.fit(X_train, y_train)

prob_test = model.predict_proba(X_test)[:, 1]

prob_ex = model.predict_proba(X_ex)[:, 1]

auc_test = roc_auc_score(y_test, prob_test)

auc_ex = roc_auc_score(y_ex, prob_ex)

auc_test_ci = auc_ci_normal(auc_test, len(y_test))

auc_ex_ci = auc_ci_normal(auc_ex, len(y_ex))

# Calibrated probabilities for Brier score

calibrated_model = CalibratedClassifierCV(

estimator=model,

method="sigmoid",

cv=5

)

calibrated_model.fit(X_train, y_train)

prob_test_cal = calibrated_model.predict_proba(X_test)[:, 1]

prob_ex_cal = calibrated_model.predict_proba(X_ex)[:, 1]

brier_test = brier_score_loss(y_test, prob_test_cal)

brier_ex = brier_score_loss(y_ex, prob_ex_cal)

return {

"Class imbalance strategy": model_name,

"Internal validation AUC (95% CI)": f"{auc_test:.3f} ({auc_test_ci[0]:.3f}-{auc_test_ci[1]:.3f})",

"Internal Brier score": round(brier_test, 4),

"External validation AUC (95% CI)": f"{auc_ex:.3f} ({auc_ex_ci[0]:.3f}-{auc_ex_ci[1]:.3f})",

"External Brier score": round(brier_ex, 4)

}

rf_params = dict(

n_estimators=516,

random_state=32,

max_depth=3,

min_samples_leaf=4,

min_samples_split=2,

criterion="gini"

)

rf_class_weight = RandomForestClassifier(

**rf_params,

class_weight="balanced"

)

rf_no_correction = RandomForestClassifier(

**rf_params,

class_weight=None

)

rf_smote = ImbPipeline(steps=[

("smote", SMOTE(random_state=42)),

("rf", RandomForestClassifier(

**rf_params,

class_weight=None

))

])

rf_undersampling = ImbPipeline(steps=[

("undersampling", RandomUnderSampler(random_state=42)),

("rf", RandomForestClassifier(

**rf_params,

class_weight=None

))

])

rf_sensitivity_results = pd.DataFrame([

evaluate_rf_sensitivity("Class weighting", rf_class_weight),

evaluate_rf_sensitivity("No imbalance correction", rf_no_correction),

evaluate_rf_sensitivity("SMOTE oversampling", rf_smote),

evaluate_rf_sensitivity("Random undersampling", rf_undersampling)

])

print(rf_sensitivity_results)

rf_sensitivity_results.to_csv(

"rf_imbalance_sensitivity_results.csv",

index=False

)

# =========================

# RF calibration plots

# =========================

from sklearn.calibration import CalibratedClassifierCV, calibration_curve

from sklearn.metrics import brier_score_loss

import matplotlib.pyplot as plt

plt.rcParams["pdf.fonttype"] = 42

plt.rcParams["ps.fonttype"] = 42

plt.rcParams["font.family"] = "Arial"

plt.rcParams["font.size"] = 11

rf_for_calibration = RandomForestClassifier(

n_estimators=516,

random_state=32,

class_weight="balanced",

max_depth=3,

min_samples_leaf=4,

min_samples_split=2,

criterion="gini"

)

rf_calibrated = CalibratedClassifierCV(

estimator=rf_for_calibration,

method="sigmoid",

cv=5

)

rf_calibrated.fit(X_train, y_train)

prob_test_rf_cal = rf_calibrated.predict_proba(X_test)[:, 1]

prob_ex_rf_cal = rf_calibrated.predict_proba(X_ex)[:, 1]

def save_calibration_plot(y_true, y_prob, title, filename, n_bins=5):

prob_true, prob_pred = calibration_curve(

y_true,

y_prob,

n_bins=n_bins,

strategy="quantile"

)

brier = brier_score_loss(y_true, y_prob)

fig, ax = plt.subplots(figsize=(5, 5))

ax.plot(

prob_pred,

prob_true,

marker="o",

linewidth=2,

markersize=6,

label="RF model"

)

ax.plot(

[0, 1],

[0, 1],

linestyle="--",

linewidth=1.5,

label="Ideal"

)

ax.text(

0.05,

0.92,

f"Brier score = {brier:.4f}",

transform=ax.transAxes,

fontsize=11,

verticalalignment="top"

)

ax.set_xlabel("Predicted probability")

ax.set_ylabel("Observed probability")

ax.set_title(title)

ax.set_xlim(0, 1)

ax.set_ylim(0, 1)

ax.legend(frameon=False, loc="lower right")

fig.tight_layout()

fig.savefig(filename, format="pdf", dpi=300, bbox_inches="tight")

plt.show()

save_calibration_plot(

y_true=y_test,

y_prob=prob_test_rf_cal,

title="Internal Validation",

filename="RF_calibration_internal.pdf"

)

save_calibration_plot(

y_true=y_ex,

y_prob=prob_ex_rf_cal,

title="External Validation",

filename="RF_calibration_external.pdf"

)

Supplementary Table S1. Missing values of variables in the train and internal validation cohorts.

| Varialbes | Percentage of missing, n (%) |
| --- | --- |
| White blood cell | 148 (6.2) |
| Red cell distribution width | 92 (3.9) |

Supplementary Table S2. Baseline characteristics of the selected predictors and outcome across the training, internal validation, and external validation cohorts.

|  | Training cohort | Internal validation cohort | External validation cohort | P |
| --- | --- | --- | --- | --- |
| N | 1668 | 715 | 789 |  |
| Selected predictors |  |  |  |  |
| Age, years | 53.8 (45.0, 63.1) | 54.0 (44.9, 63.9) | 58.0 (47.1, 65.9) | < 0.001 |
| Systolic blood pressure, mmHg | 120.0 (110.0, 136.0) | 120.0 (110.0, 136.0) | 124.0 (110.0, 140.0) | 0.021 |
| Coronary artery disease, n (%) | 33 (2.0) | 15 (2.1) | 31 (3.9) | 0.011 |
| Left ventricular hypertrophy, n (%) | 134 (8.0) | 68 (9.5) | 56 (7.1) | 0.227 |
| Transferrin, mg/dL | 272.0 (242.0, 303.0) | 272.0 (245.0, 302.5) | 270.0 (244.0, 304.0) | 0.879 |
| Triglycerides, mg/dL | 96.0 (68.0, 143.0) | 101.0 (72.0, 151.0) | 106.0 (75.0, 161.0) | < 0.001 |
| HDL-C, mg/dL | 47.0 (39.0, 55.0) | 46.0 (38.0, 55.0) | 45.0 (38.0, 54.0) | 0.075 |
| LDL-C, mg/dL | 132.0 (107.0, 163.0) | 135.0 (110.0, 163.0) | 141.0 (112.0, 173.0) | < 0.001 |
| Apolipoprotein A1, g/L | 1.3 (1.1, 1.5) | 1.3 (1.2, 1.5) | 1.3 (1.1, 1.5) | 0.033 |
| Apolipoprotein B, g/L | 0.9 (0.7, 1.1) | 0.9 (0.8, 1.2) | 0.9 (0.8, 1.2) | 0.002 |
| Outcome |  |  |  |  |
| Sudden cardiac death, n (%) | 37 (2.2) | 16 (2.2) | 21 (2.7) | 0.779 |

Abbreviations: HDL-C, high-density lipoprotein cholesterol; LDL-C, low-density lipoprotein cholesterol.

Supplementary Table S3. Best hyperparameters of each machine learning model

| Classifiers | Hyperparameters | |
| --- | --- | --- |
| Light Gradient Boosting Machine | n_estimators | 54 |
|  | max_depth | 5 |
|  | learning_rate | 0.005 |
|  | boosting_type | Gbdt |
|  | objective | Binary |
|  | num_leaves | 31 |
|  | colsample_bytree | 0.8 |
|  | min_child_samples | 195 |
|  | subsample | 0.9 |
|  | class_weight | ‘balanced’ |
|  |  |  |
| Random Forest | n_estimators | 516 |
|  | max_depth | 3 |
|  | criterion | ‘gini’ |
|  | min_samples_leaf | 4 |
|  | random_state | 32 |
|  | class_weight | ‘balanced’ |
|  |  |  |
| Logistic Regression | C | 0.5 |
|  | penalty | l2 |
|  | solver | ‘sag’ |
|  | max_iter | 200 |
|  | random_state | 5 |
|  | class_weight | ‘balanced’ |
|  |  |  |
| Support Vector Machine | C | 0.4 |
|  | kernel | ‘rbf’ |
|  | probability | True |
|  | tol | 0.0001 |
|  | gamma | ‘auto’ |
|  | class_weight | ‘balanced’ |
|  |  |  |
| Multilayer Perceptron | solver | ‘adam’ |
|  | activation | ‘relu’ |
|  | hidden_layer_sizes | (2, 4) |
|  | alpha | 0.0001 |
|  | max_iter | 500 |
|  | random_state | 3 |
|  |  |  |
| K-Nearest Neighbors | n_neighbors | 25 |
|  | p | 5 |
|  | metric | chebyshev |
|  | weights | distance |

Supplementary Table S4. DeLong test for AUC differences between machine learning models and traditional risk scores in the internal and external validation cohorts.

| Model | AUC | ΔAUC | Z statistic | DeLong test P |
| --- | --- | --- | --- | --- |
| Internal Validation Cohort |  |  |  |  |
| Framingham Risk Score | 0.737 | *Reference* | | |
| Cardiovascular Risk PROCAM Score | 0.776 | 0.039 | 1.196 | 0.232 |
| Light Gradient Boosting Machine | 0.766 | 0.029 | 0.691 | 0.490 |
| Random Forest | 0.824 | 0.087 | 2.206 | 0.027 |
| Logistic Regression | 0.745 | 0.008 | 0.151 | 0.880 |
| Support Vector Machine | 0.778 | 0.041 | 0.672 | 0.502 |
| Multilayer Perceptron | 0.774 | 0.036 | 0.571 | 0.568 |
| K-Nearest Neighbours | 0.729 | -0.008 | -0.094 | 0.925 |
|  |  |  |  |  |
| External Validation Cohort |  |  |  |  |
| Framingham Risk Score | 0.733 | Reference | | |
| Cardiovascular Risk PROCAM Score | 0.755 | 0.022 | 0.660 | 0.509 |
| Light Gradient Boosting Machine | 0.689 | -0.044 | -0.624 | 0.533 |
| Random Forest | 0.815 | 0.082 | 2.665 | 0.008 |
| Logistic Regression | 0.699 | -0.035 | -0.676 | 0.499 |
| Support Vector Machine | 0.708 | -0.026 | -0.479 | 0.632 |
| Multilayer Perceptron | 0.637 | -0.096 | -1.702 | 0.089 |
| K-Nearest Neighbours | 0.611 | -0.122 | -1.711 | 0.087 |

Abbreviations: AUC, area under the curve.

Supplementary Table S5. Performance metrics of the final random forest model with 95% confidence intervals.

| Model | Metric | Internal validation | External validation |
| --- | --- | --- | --- |
| LightGBM | AUC | 0.766 (0.735-0.797) | 0.689 (0.657-0.721) |
|  | Accuracy | 0.641 (0.605-0.675) | 0.573 (0.538-0.607) |
|  | Specificity | 0.635 (0.599-0.670) | 0.569 (0.534-0.604) |
|  | Precision | 0.052 (0.031-0.085) | 0.043 (0.026-0.070) |
|  | Recall | 0.875 (0.640-0.965) | 0.714 (0.500-0.862) |
|  | F1-score | 0.098 (0.051-0.151) | 0.082 (0.046-0.119) |
|  | G-mean | 0.213 (0.142-0.276) | 0.176 (0.116-0.233) |
|  | Brier score | 0.0217 | 0.0257 |
|  |  |  |  |
| Random forest | AUC | 0.824 (0.796-0.852) | 0.815 (0.788-0.842) |
|  | Accuracy | 0.912 (0.889-0.931) | 0.880 (0.855-0.900) |
|  | Specificity | 0.923 (0.901-0.940) | 0.893 (0.869-0.913) |
|  | Precision | 0.115 (0.057-0.218) | 0.089 (0.046-0.166) |
|  | Recall | 0.438 (0.231-0.668) | 0.381 (0.208-0.591) |
|  | F1-score | 0.182 (0.071-0.308) | 0.144 (0.061-0.232) |
|  | G-mean | 0.224 (0.098-0.352) | 0.184 (0.081-0.288) |
|  | Brier score | 0.0211 | 0.0249 |
|  |  |  |  |
| Logistic regression | AUC | 0.745 (0.713-0.777) | 0.699 (0.667-0.731) |
|  | Accuracy | 0.048 (0.034-0.066) | 0.039 (0.028-0.055) |
|  | Specificity | 0.026 (0.016-0.040) | 0.013 (0.007-0.024) |
|  | Precision | 0.023 (0.014-0.037) | 0.027 (0.018-0.041) |
|  | Recall | 1.000 (0.806-1.000) | 1.000 (0.845-1.000) |
|  | F1-score | 0.045 (0.023-0.067) | 0.052 (0.033-0.074) |
|  | G-mean | 0.152 (0.108-0.186) | 0.164 (0.129-0.196) |
|  | Brier score | 0.0215 | 0.0256 |
|  |  |  |  |
| SVM | AUC | 0.778 (0.747-0.808) | 0.708 (0.676-0.739) |
|  | Accuracy | 0.806 (0.775-0.833) | 0.777 (0.747-0.805) |
|  | Specificity | 0.811 (0.780-0.838) | 0.784 (0.753-0.812) |
|  | Precision | 0.064 (0.034-0.117) | 0.062 (0.035-0.108) |
|  | Recall | 0.562 (0.332-0.769) | 0.524 (0.324-0.717) |
|  | F1-score | 0.115 (0.051-0.192) | 0.111 (0.053-0.170) |
|  | G-mean | 0.189 (0.102-0.285) | 0.180 (0.099-0.259) |
|  | Brier score | 0.0215 | 0.0256 |
|  |  |  |  |
| MLP | AUC | 0.773 (0.742-0.804) | 0.637 (0.604-0.671) |
|  | Accuracy | 0.639 (0.603-0.674) | 0.575 (0.541-0.609) |
|  | Specificity | 0.634 (0.597-0.669) | 0.572 (0.536-0.606) |
|  | Precision | 0.052 (0.031-0.085) | 0.044 (0.027-0.071) |
|  | Recall | 0.875 (0.640-0.965) | 0.714 (0.500-0.862) |
|  | F1-score | 0.082 (0.045-0.123) | 0.098 (0.054-0.152) |
|  | G-mean | 0.213 (0.144-0.276) | 0.176 (0.116-0.234) |
|  | Brier score | 0.0216 | 0.0258 |
|  |  |  |  |
| KNN | AUC | 0.729 (0.697-0.762) | 0.611 (0.577-0.645) |
|  | Accuracy | 0.676 (0.640-0.709) | 0.631 (0.597-0.664) |
|  | Specificity | 0.678 (0.643-0.712) | 0.637 (0.602-0.670) |
|  | Precision | 0.038 (0.020-0.071) | 0.031 (0.017-0.058) |
|  | Recall | 0.562 (0.332-0.769) | 0.429 (0.245-0.635) |
|  | F1-score | 0.072 (0.031-0.125) | 0.058 (0.023-0.098) |
|  | G-mean | 0.147 (0.073-0.221) | 0.116 (0.050-0.178) |
|  | Brier score | 0.0218 | 0.0258 |

95% confidence intervals for AUC were estimated using normal approximation; confidence intervals for proportion-based metrics were estimated using Wilson intervals; confidence intervals for F- score and G-mean were estimated using 1,000 bootstrap resamples. Brier scores are presented as point estimates.

Abbreviations: AUC, area under the receiver operating characteristic curve; CI, confidence interval; KNN, K-Nearest Neighbours; LightGBM, Light Gradient Boosting Machine; MLP, Multilayer Perceptron; SVM, Support Vector Machine..

Supplementary Table S6. Sensitivity analysis of the random forest model using alternative class imbalance handling strategies.

| Class imbalance strategy | Internal validation AUC (95% CI) | Internal Brier score | External validation AUC (95% CI) | External Brier score |
| --- | --- | --- | --- | --- |
| Class weighting (primary analysis) | 0.824 (0.796-0.852) | 0.0211 | 0.815 (0.788-0.842) | 0.0249 |
| No imbalance correction | 0.794 (0.651-0.915) | 0.0194 | 0.829 (0.734-0.915) | 0.0234 |
| SMOTE oversampling | 0.712 (0.623-0.799) | 0.1978 | 0.708 (0.614-0.796) | 0.2160 |
| Random undersampling | 0.789 (0.659-0.900) | 0.2147 | 0.774 (0.647-0.895) | 0.2217 |

The primary analysis used class weighting to account for class imbalance. Confidence intervals for AUC were estimated using 1,000 bootstrap resamples.

Abbreviations: AUC, area under the receiver operating characteristic curve; CI, confidence interval; SMOTE, Synthetic Minority Over-sampling Technique.

Supplementary Table S7. Performance of the random forest model for time-specific sudden cardiac death prediction at 5, 10, and 15 years.

| Time horizon | Cohort | Event, n (%) | AUC (95% CI) | Accuracy | Recall | Specificity | Precision | F1 score | G-mean |
| --- | --- | --- | --- | --- | --- | --- | --- | --- | --- |
| 5-year | Internal validation | 7 (0.98) | 0.895 (0.872-0.917) | 0.987 | 0.000 | 0.997 | 0.000 | 0.000 | 0.000 |
|  | External validation | 6 (0.76) | 0.898 (0.876-0.919) | 0.984 | 0.333 | 0.989 | 0.182 | 0.235 | 0.246 |
|  |  |  |  |  |  |  |  |  |  |
| 10-year | Internal validation | 11 (1.54) | 0.932 (0.913-0.950) | 0.983 | 0.273 | 0.994 | 0.429 | 0.333 | 0.342 |
|  | External validation | 8 (1.01) | 0.938 (0.921-0.955) | 0.978 | 0.500 | 0.983 | 0.235 | 0.320 | 0.343 |
|  |  |  |  |  |  |  |  |  |  |
| 15-year | Internal validation | 14 (1.96) | 0.865 (0.840-0.890) | 0.958 | 0.429 | 0.969 | 0.214 | 0.286 | 0.303 |
|  | External validation | 15 (1.90) | 0.856 (0.831-0.880) | 0.943 | 0.467 | 0.952 | 0.159 | 0.237 | 0.272 |

Time-specific sudden cardiac death outcomes were defined as events occurring within 5, 10, or 15 years from baseline. The same predefined random forest architecture as the primary analysis was applied for all time-horizon analyses.

Abbreviations: AUC, area under the receiver operating characteristic curve; CI, confidence interval.

Supplementary Table S8. Incremental discrimination improvement and DeLong comparisons between nested models.

| Model | Internal validation | | | External validation | | |
| --- | --- | --- | --- | --- | --- | --- |
|  | AUC (95% CI) | ΔAUC vs Model 3 | DeLong P | AUC (95% CI) | ΔAUC vs Model 3 | DeLong P |
| Model 1 | 0.719 (0.686-0.751) | 0.105 | 0.008 | 0.749 (0.718-0.779) | 0.066 | 0.197 |
| Model 2 | 0.742 (0.710-0.774) | 0.082 | 0.147 | 0.697 (0.665-0.729) | 0.118 | 0.025 |
| Model 3 | 0.824 (0.796-0.852) | Reference | Reference | 0.815 (0.788-0.842) | Reference | Reference |

Model 1 included clinical variables only (Age, SBP, CAD, LVH, and Total Ferritin). Model 2 included Model 1 variables plus conventional lipid markers (Triglyceride, HDL-C, and LDL-C). Model 3 was the full model incorporating Model 2 variables plus advanced lipid biomarkers (Apolipoprotein A1 and Apolipoprotein B). ΔAUC was calculated relative to Model 3. DeLong tests compared each model against Model 3 as the reference.

Abbreviations: AUC, area under the receiver operating characteristic curve; CAD, coronary artery disease; CI, confidence interval; HDL-C, high-density lipoprotein cholesterol; LDL-C, low-density lipoprotein cholesterol; LVH, left ventricular hypertrophy; RF, random forest; SBP, systolic blood pressure.

Supplementary Table S9. Optimal probability threshold and predictive performance of the Random Forest.

|  | Optimal threshold | AUC | Sensitivity | Specificity | PPV | NPV |
| --- | --- | --- | --- | --- | --- | --- |
| Internal validation cohort | 0.37 | 0.824 | 0.875 | 0.654 | 0.055 | 0.996 |
| External validation cohort | 0.37 | 0.815 | 0.857 | 0.598 | 0.054 | 0.993 |

Abbreviations: AUC, area under the curve; NPV, negative predictive value; PPV, positive predictive value.


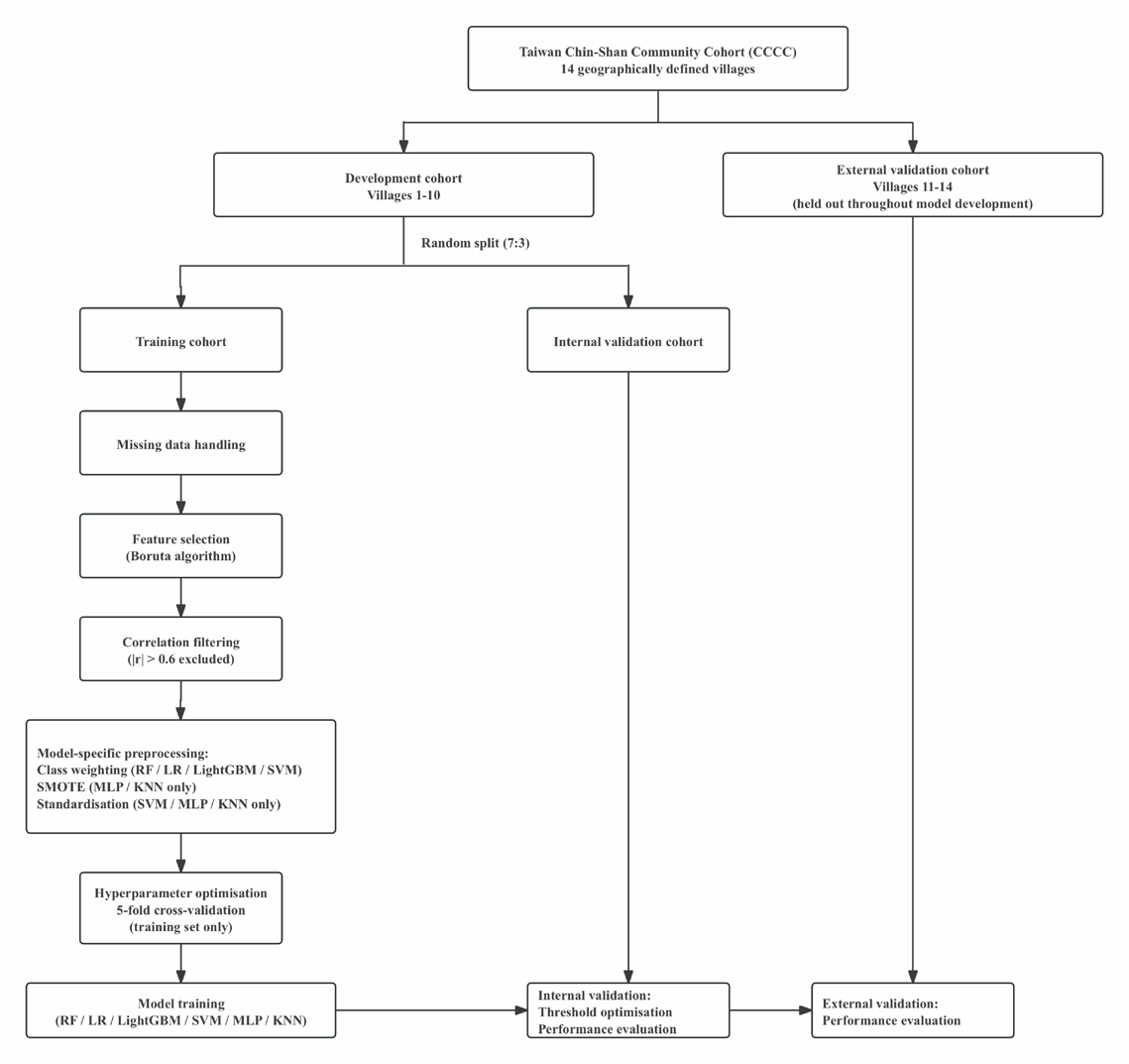


Supplementary Figure S1. Schematic workflow of machine learning model development, validation, and preprocessing procedures. The Taiwan Chin-Shan Community Cohort (CCCC) was geographically partitioned into a development cohort (villages 1–10) and an independent external validation cohort (villages 11–14), which was held out throughout model development. The development cohort was randomly divided into training and internal validation sets in a 7:3 ratio. Missing data handling, feature selection using the Boruta algorithm, correlation filtering, model-specific preprocessing (including class weighting, SMOTE where applicable, and standardisation for selected models), hyperparameter optimisation, and model training were performed within the training cohort only to minimise information leakage. The internal validation cohort was used for threshold optimisation and performance evaluation, and the final model was subsequently assessed in the geographically distinct external validation cohort. CCCC, Taiwan Chin-Shan Community Cohort; KNN, K-Nearest Neighbours; LightGBM, Light Gradient Boosting Machine; LR, Logistic Regression; MLP, Multilayer Perceptron; RF, Random Forest; SMOTE, Synthetic Minority Over-sampling Technique; SVM, Support Vector Machine.


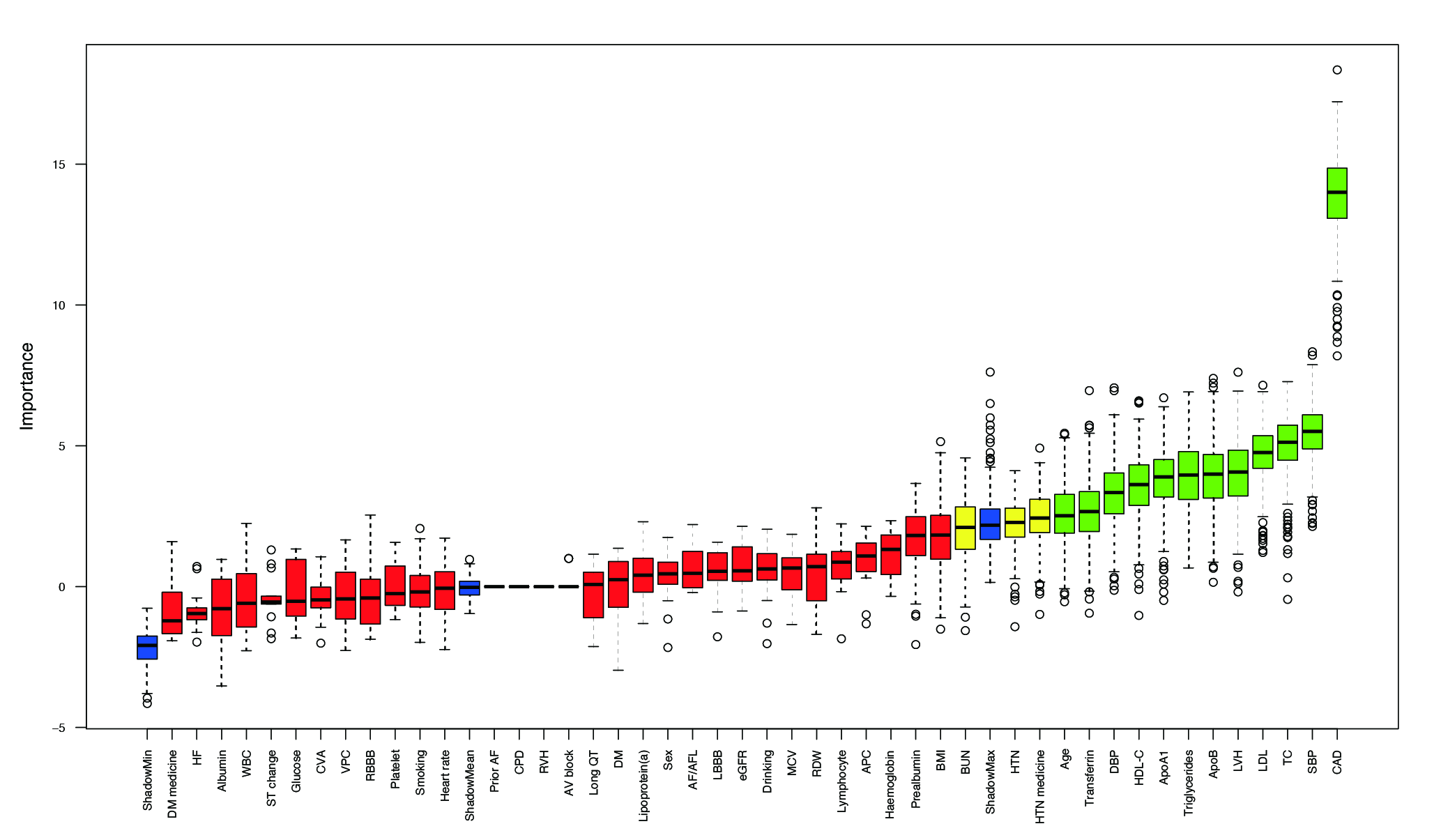


**Supplementary Figure S2 Boruta feature importance ranking for predicting SCD in the training cohort.** AF, atrial fibrillation; AFL, atrial flutter; APC, atrial premature contraction; ApoA1, apolipoprotein A1; ApoB, apolipoprotein B; AV, atrioventricular; BMI, body mass index; BUN, blood urea nitrogen; CAD, coronary artery disease; CPD, chronic pulmonary disease; CVA, cerebrovascular accident; DBP, diastolic blood pressure; DM, diabetes mellitus; eGFR, estimated glomerular filtration rate; HDL-C, high-density lipoprotein cholesterol; HF, congestive heart failure; HTN, hypertension; LBBB, left bundle branch block; LDL-C, low-density lipoprotein cholesterol; LVH, left ventricular hypertrophy; MCV, mean corpuscular volume; MI, myocardial infarction; RBBB, right bundle branch block; RDW, red cell distribution width; RVH, right ventricular hypertrophy; SBP, systolic blood pressure; TC, total cholesterol; VPC, ventricular premature contraction; WBC, white blood cell.


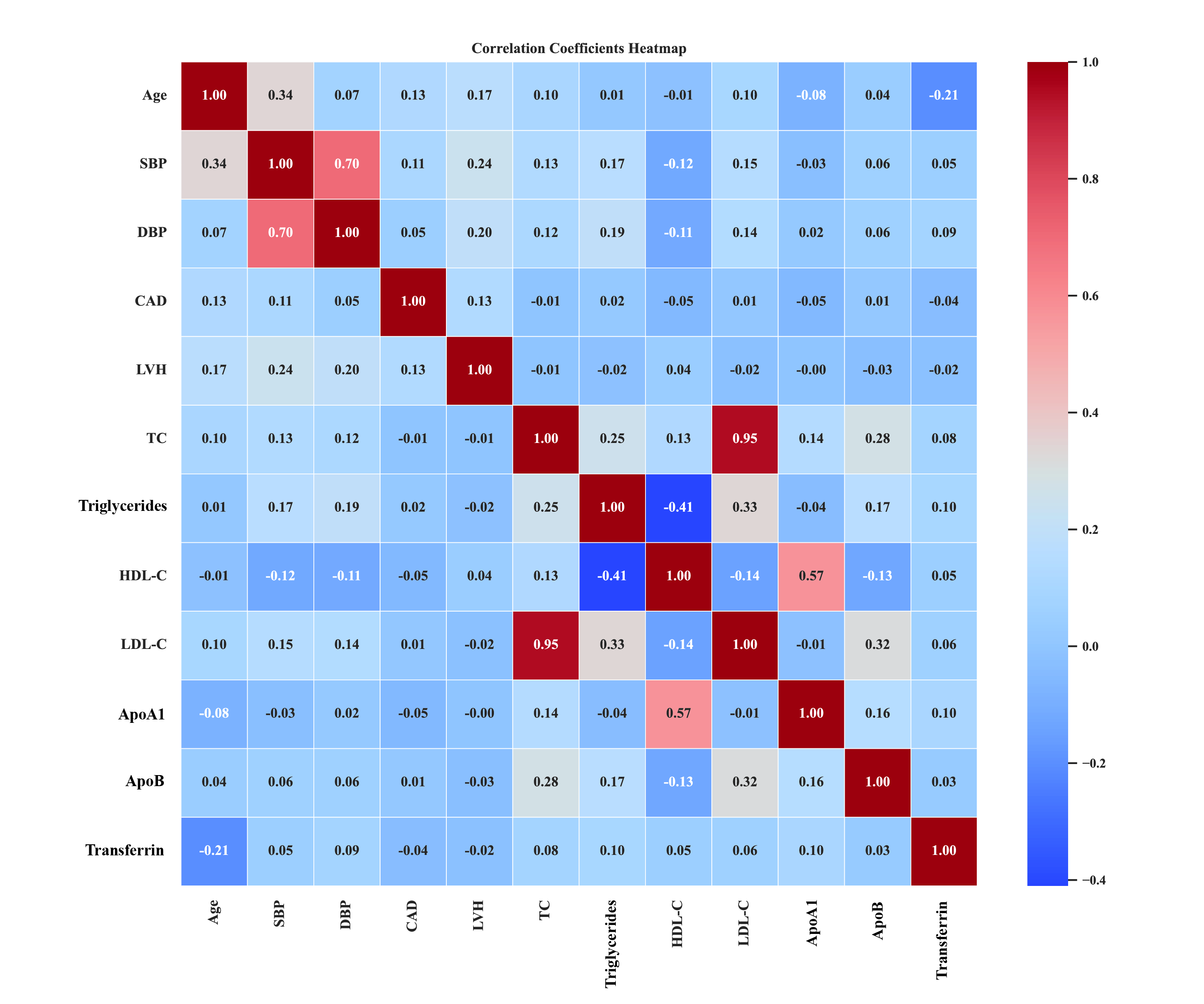


**Supplementary Figure S3 Pearson correlation heatmap showing pairwise correlations among candidate predictors selected by the Boruta algorithm.** Variables exhibiting high collinearity (absolute Pearson correlation coefficient |r| > 0.6) were considered redundant and excluded from subsequent model development.


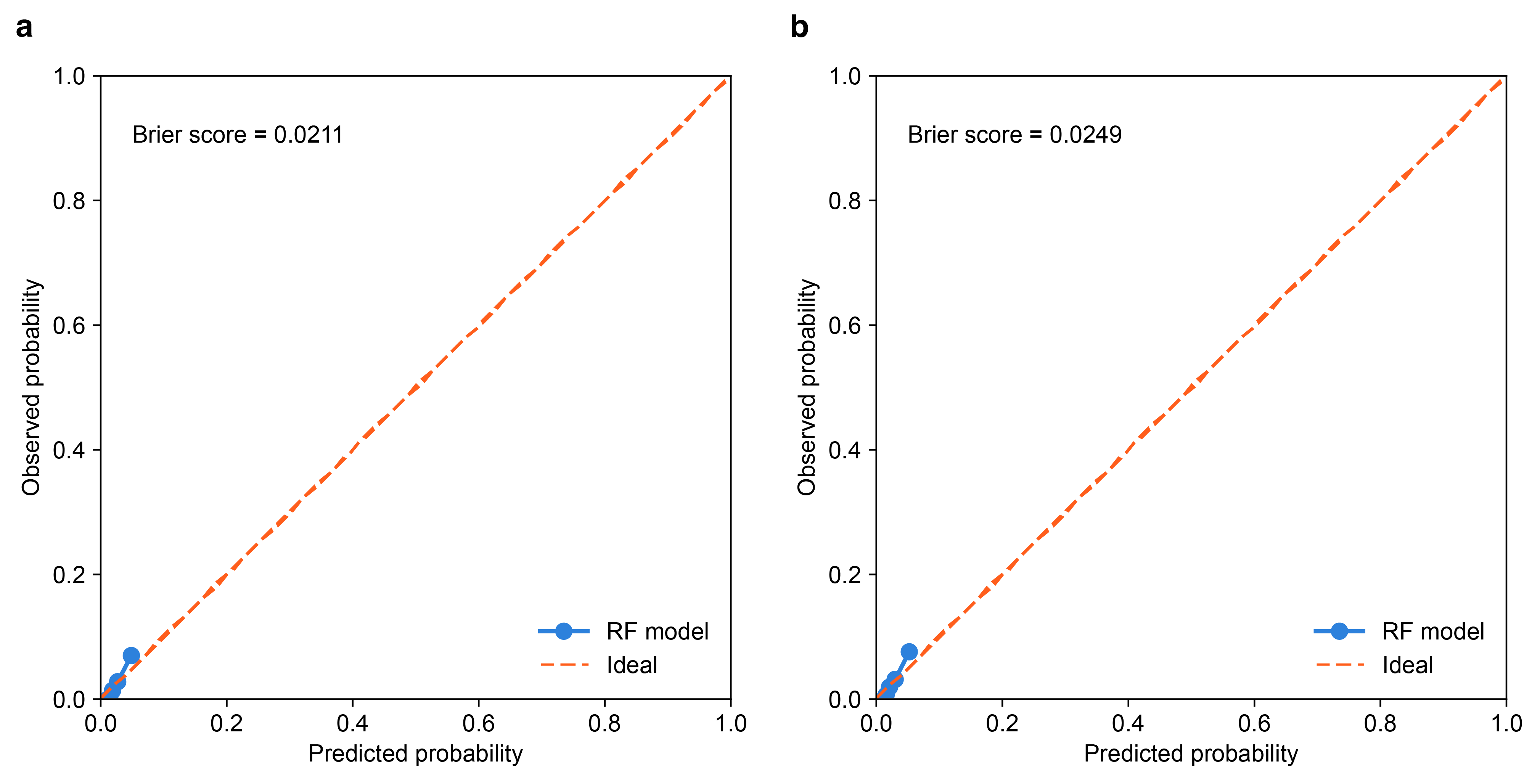


**Supplementary Figure S4. Calibration of the final random forest model.** Calibration plots for the final RF model in the internal (a) and external (b) validation cohorts. The dashed diagonal line indicates ideal agreement between predicted and observed risks. Brier scores are displayed in each panel. RF, random forest.
